# Uridine cytidine kinases govern molnupiravir bioactivation and anti-SARS-CoV-2 activity

**DOI:** 10.1371/journal.ppat.1014225

**Published:** 2026-05-29

**Authors:** Huazhang Shu, Julian M. Ludäscher, Sushma Sharma, Seher Alam, Lilian Frank, Emma Scaletti Hutchinson, Marianna Tampere, Chloé Lévêque, André B.P. van Kuilenburg, Nicholas C.K. Valerie, Mikael Altun, Andrei Chabes, Pål Stenmark, Sean G. Rudd, Si Min Zhang

**Affiliations:** 1 Department of Oncology-Pathology, Science for Life Laboratory (SciLifeLab), Karolinska Institutet, Stockholm, Sweden; 2 Department of Biochemistry and Biophysics, Stockholm University, Stockholm, Sweden; 3 Department of Medical Biochemistry and Biophysics, Umeå University, Umeå, Sweden; 4 Department of Laboratory Medicine, Division of Clinical Physiology, Karolinska Institutet Huddinge, Huddinge, Sweden; 5 Department of Laboratory Medicine, Division of Clinical Physiology, Karolinska University Hospital, Huddinge, Sweden; 6 Department of Oncology-Pathology, Precision Cancer Medicine, Karolinska Institutet, Science for Life Laboratory, Solna, Sweden; 7 Department of Microbiology, Tumor and Cell Biology (MTC), Karolinska Institutet, Stockholm, Sweden; 8 Amsterdam Gastroenterology Endocrinology Metabolism, Amsterdam, The Netherlands; 9 Department of Clinical Chemistry, Amsterdam UMC, Vrije Universiteit Amsterdam, Laboratory Genetic Metabolic Diseases, Amsterdam, The Netherlands; Washington University School of Medicine in Saint Louis: Washington University in St Louis School of Medicine, UNITED STATES OF AMERICA

## Abstract

Molnupiravir is a nucleoside analogue antiviral drug against RNA viruses, including its clinical indication SARS-CoV-2. Whilst its mechanism-of-action is well defined, host factors that regulate its therapeutic responses have not been thoroughly deciphered and characterized. Here we show that uridine cytidine kinases (UCKs), key enzymes in pyrimidine salvage, effectively phosphorylate and thereby bioactivate N4-hydroxycytidine (NHC) – the active compound of molnupiravir, thus dictating its anti-SARS-CoV-2 efficacy and furthermore selectivity. In vitro, both isoforms of UCKs (UCK1 and UCK2) effectively phosphorylated NHC, where the structural basis of the catalysis was further deciphered via the first complete substrate bound co-crystal structure of UCK, i.e., UCK1-NHC-AMPPNP. In SARS-CoV-2-infected cells, UCK2 knockdown via siRNA hampered the intracellular accumulation of the tri-phosphorylated antiviral metabolite of NHC, resulting in a 10-fold reduction of the antiviral efficacy, and surprisingly, 2-fold reduction of its selectivity, which were critically recapitulated in a dose-dependent manner using a pan-UCK inhibitor. Altogether, this work underscores UCKs as pivotal players in upholding molnupiravir efficacy and therapeutic window, and furthermore as pharmacologically tractable targets for tailoring the drug response.

## Author summary

Coronavirus outbreaks, including the SARS outbreak in the early 2000s and the more recent COVID-19 pandemic, have posed a significant threat to global public health. Molnupiravir, a drug originally developed to treat a range of RNA virus infections, was quickly repurposed and approved for COVID-19, aiding patient recovery. However, the reasons why this drug works better in some patients than others are not well understood. In our work, we found that two human enzymes, called uridine cytidine kinases (UCKs), play a key role in activating molnupiravir once it enters cells. These enzymes convert the drug into its active form, which is necessary for blocking viral replication. We showed that reducing the level of one of these enzymes, UCK2, in infected cells led to a clear drop in the drug’s antiviral activity and reduced its ability to selectively target the virus over the host. We confirmed these findings using a chemical inhibitor that blocks these enzymes. By directly examining the structure of molnupiravir-bound UCK, we gained insight into how this activation process occurs. Overall, our findings highlight the importance of host cell enzymes e.g. UCKs in determining how well molnupiravir works and suggest new ways to improve this antiviral treatment.

## Introduction

**Fig 1:**
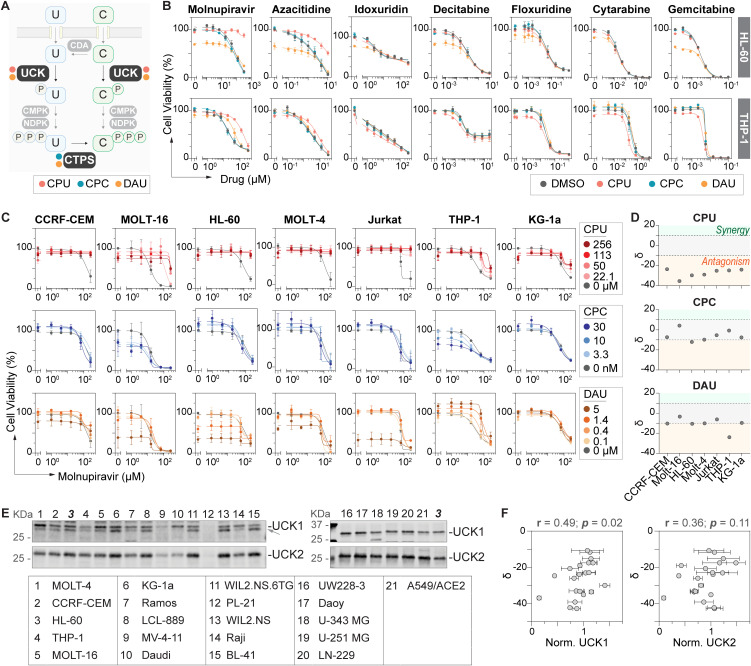
UCK inhibition limits cellular response to molnupiravir and its cell-active metabolite NHC. **A.** Schematic representation of cellular roles of UCK in pyrimidine salvage pathway. UCKs govern the first- and rate-limiting step of pyrimidine salvage pathway. It catalyzes the mono-phosphorylation of uridine and cytidine into uridine monophosphate (UMP) and cytidine monophosphate (CMP), respectively, which upon subsequent phosphorylation by CMPK and NDPK are converted to the tri-phosphorylated nucleotides. Cytidine triphosphate (CTP) could be further converted from uridine triphosphate (UTP) by CTPS. In the cell viability-based screening, UCK inhibitor CPU, UCK/CTPS dual inhibitor DAU, and finally CTPS inhibitor CPC were employed, as highlighted in the schematic representation. UCK, uridine cytidine kinase 1/2; CTPS, CTP synthase 1/2; CMPK, cytidine monophosphate kinase; CDA, cytidine deaminase; NDPK, nucleoside-diphosphate kinase; CPU, cyclopentenyl uracil; DAU, 3-Deazauridine; CPC, cyclopentenyl cytosine. **B.** Focused nucleoside analogue drug library screening identified UCK as a limiting factor for the cellular response of molnupiravir. AML HL-60 and THP1 cells were treated with nucleoside analogues drugs in the presence or absence of 100 µM CPU, 12 nM CPC, and 1 µM DAU for 96 hours before cell viabilities were determined using a resazurin reduction assay. Relative viability percentages were determined by normalizing background-subtracted fluorescence signals at 584 nm to DMSO controls. In both HL-60 and THP1 cells, inhibition of UCK by CPU and DAU, but not CTPS inhibition by CPC, retarded the cytotoxicity of molnupiravir and azacitidine. **C-D.** Inhibition of UCK consistently antagonized NHC, metabolite of molnupiravir, in multiple cell lines. In C, relative cell viability curves in multiple leukemia cell lines treated with NHC, in the presence or absence of CPU, CPC, or DAU; in D, the relative cell viability data as presented in C were further used to determine synergy scores (δ), calculated using SynergyFinder. A δ > 10 reflects synergy, and a δ < -10 reflects antagonism. **E-F.** Cellular UCK levels positively correlated with CPU-induced NHC antagonism. In E, Western blot analysis of UCK expression levels in multiple cell lines, where lysates containing equal amount of protein were separated by SDS-PAGE, probed with UCK1 and UCK2 specific antibodies, and then subject to densitometry analysis. UCK1 and UCK2 expressions in the cell lines were normalized to the levels in HL-60 cells, and were presented as relative levels. In F, cell lines in E were subjected to resazurin reduction assay upon 96-hour treatment with NHC or DMSO control, in the presence or absence of CPU. Synergy scores were subsequently determined based on the cell viability data, as described in D, and further demonstrated positive correlations with both UCK1 and UCK2 expression levels. r and p values of Spearman correlation analysis are shown.

Uridine and cytidine kinase (UCK) (EC 2.7.1.48) is a pyrimidine ribonucleoside kinase with two isoforms sharing ~70% sequence homology. Isoform 1 (UCK1) is uniformly expressed across tissues, whilst the catalytically superior isoform 2 (UCK2) is primarily reserved for embryonic and placental tissues, but can be re-expressed in malignancies [1,2]. Physiologically, UCKs govern the first and rate-limiting step of the pyrimidine salvage pathway by catalyzing the mono-phosphorylation of plasma-derived uridine and cytidine into uridine and cytidine monophosphates (UMP and CMP), respectively. UMP and CMP can then be sequentially phosphorylated by CMP kinase (CMPK) and nucleoside-diphosphate kinase (NDPK) into uridine triphosphate (UTP) and cytidine triphosphate (CTP), fueling cellular demand for pyrimidine nucleotides (Fig 1A) [3].

Nucleotide salvage pathways are also central for the activation of nucleoside analogue (NA) therapeutics, which constitute an important class of anticancer and antiviral agents. NA drugs require stepwise phosphorylation to generate their active metabolites, typically their triphosphate (NA-TP) forms, which can then compete with their physiological counterparts to exert their therapeutic effects. Similar to its physiological activity, UCKs have been shown to mono-phosphorylate several uridine and cytidine analogues including azacitidine and 5-fluorouridine and the investigational agent RX-3117, and thereby control the accumulation of the active NA-TP pool [1,4,5]. Furthermore, low UCK expression and loss-of-activity mutations have been associated with resistance to azacitidine, which can be restored upon UCK re-expression [6–8]. Whilst these studies support UCKs as putative biomarkers for azacitidine therapy, the broader role of UCKs in governing the efficacy of NA-based therapies, and particularly newly approved NA drugs, such as the severe acute respiratory coronavirus 2 (SARS-CoV-2) therapy molnupiravir [9], remains unclear.

Molnupiravir is the isopropyl ester pro-drug of β-D-N4-hydroxycytidine (NHC), a broad-spectrum antiviral with activities against multiple RNA viruses aside from its clinical indication SARS-CoV-2, including chikungunya virus [10], hepatitis C virus [11], influenza viruses [12], Ebola virus [13], and other coronavirus family members including SARS-CoV-1 [14]. Upon administration, molnupiravir is rapidly converted to NHC by plasma esterase, which is then taken up into cells and subsequently phosphorylated to NHC triphosphate (NHC-TP). As a close analogue of CTP, NHC-TP is readily mis-incorporated into the viral genome by the viral RNA polymerase, and is then ambiguously matched with G or A in subsequent rounds of replication, resulting in defective virions with lethal mutation burden and abolished infectivity [15]. Aside from introducing mutations in the viral genome, several studies have also shown that molnupiravir/NHC have off-target effects on host cells, through inducing mutations in the host cell genome in a similar fashion [16,17], and/or in a mutagenesis-independent manner, e.g., oxidative stress [18,19]. In particular, a study by Xu et al utilizing genome-wide CRISPR/Cas9 screening has identified that UCK, specifically UCK2, is critical to the host mutagenesis activity of molnupiravir/NHC, potentially through promoting the phosphorylation of NHC into its mutagenic nucleotide forms [17]. However, no direct and mechanistic characterization of UCKs, either UCK1 or UCK2, in the bioactivation of NHC and subsequent modulation of the antiviral therapeutic efficacy/selectivity have been reported.

In light of the translational potential of UCKs as biomarkers for NA therapy, we set out to systematically profile NA drugs phosphorylated and thereby activated by UCKs using a cell viability-based screening platform. We then identified NHC/molnupiravir as a potential substrate of UCKs. Subsequent target engagement and enzymatic kinetics studies confirmed that UCKs could effectively phosphorylate NHC in vitro, with the structural basis of the catalysis elucidated through a X-ray co-crystal structure of UCK1 in complex with NHC and ATP – the first complete substrate-bound structure of UCKs. In cells, both UCK knockdown via siRNA and inhibition via cyclopentenyl uracil (CPU) effectively limited the accumulation of the active metabolite NHC-TP, resulting in a reduction of the anti-SARS-CoV-2 efficacy by 10-fold, and unexpectedly, a significantly narrowed drug selectivity. Collectively, these data mechanistically establish the pivotal role of UCKs as the first step of NHC bioactivation and thereby the abilities of these enzymes to control drug efficacy and therapeutic window, and furthermore, underscore their potential as a viable target for tailoring NA drug response.

## Results

### UCKs dictate molnupiravir response in a cell viability-based screening platform

Previous independent studies have shown that UCKs can bioactivate several NA oncology drugs. Here we initiated the study by systematically profiling NA drugs phosphorylated and thereby activated by UCKs, focusing on newly approved and emerging therapeutics with indications for oncology and/or viral infection. Firstly, a structure-based *in silico* screening of 3323 compounds was performed, resulting in a focused group of 27 compounds as potential substrates of UCKs. Albeit being optimized for selectivity index, antiviral NA drugs could cause cytotoxicity at high concentrations, which often directly correlates to the cellular levels of their triphosphate active metabolites [20]. Based on this observation, for the in vitro validation of our *in silico* screening output – either anti-viral or -cancer applications – we conducted a cell viability-based screening, where involvement of UCK in drug activation was assessed based on drug-induced cytotoxicity (Fig 1A). Specifically, acute myeloid leukemia cell lines HL-60 and THP-1 were treated with increasing concentrations of screening drugs for 96 hours before cell viability was determined by resazurin reduction assay, done in the presence or absence of high doses of previously reported cell-active inhibitors of UCKs - cyclopentenyl uracil (CPU) [21] or 3-deazauridine (DAU) [22,23]. Aside from UCKs, DAU primarily targets CTP synthase (CTPS) upon intracellular tri-phosphorylation. To specifically assess the influence of UCKs on drug efficacy, the screening campaign therefore further included cyclopentenyl cytosine (CPC), a sub-micromolar CTPS inhibitor [24], as a control compound (Fig 1A).

Using this assay, we observed that UCK inhibitor CPU, and to a lesser extent UCK/CTPS dual inhibitor DAU, but not the CTPS inhibitor CPC, protected both HL-60 and THP-1 cells from azacitidine, an anti-leukemic agent known to be activated and thereby controlled by UCKs [6,7], hence validating the screening setup. Interestingly, similar rescue was observed for the anti-SARS-CoV-2 drug molnupiravir (Fig 1B). Molnupiravir is the isopropyl ester pro-drug of the phosphorylatable antiviral NHC, which is rapidly converted by plasma esterase upon administration [25]. Confirming the screening result using molnupiravir, dose-response matrices of NHC and DAU/CPU/CPC in multiple cell line models revealed that inhibition of UCKs by CPU consistently antagonized NHC-induced cytotoxicity with drug combination scores routinely below -20 (i.e., strong antagonism), which is followed by marginal antagonism by DAU but not by CPC (Fig 1C-D). These data suggest that UCKs are the principal kinases governing the intracellular responses of NHC/molnupiravir.

UCKs have two isoforms, the ubiquitously expressed UCK1 of lower catalytic efficacy and the more efficient UCK2 primarily reserved for placenta and overexpressed in cancers [1]. As the first step to delineate the principal UCK governing the cellular response of molnupiravir, we then correlated the intracellular UCK1 and UCK2 levels with CPU-induced NHC antagonism, in a panel of 21 cell lines of different tissue and disease origins (Fig 1E-F and S1 Fig). We reason that for the enzyme critical for NHC bioactivation, upon its inhibition, cells of higher enzyme level would sustain greater loss of active drug metabolites and thereafter cytotoxicity. Indeed, we observed that in the tested cell lines, CPU-induced antagonism of NHC positively correlated with UCK1 protein levels (r = 0.49; p = 0.02). A similar positive correlation, albeit not statistically significant (r = 0.36; p = 0.11), was observed with UCK2. These data collectively suggest that UCK1, and potentially UCK2, play principal roles in molnupiravir/NHC bioactivation and efficacy.

### NHC engages recombinant UCK1 and 2 in an ATP-dependent manner

**Fig 2:**
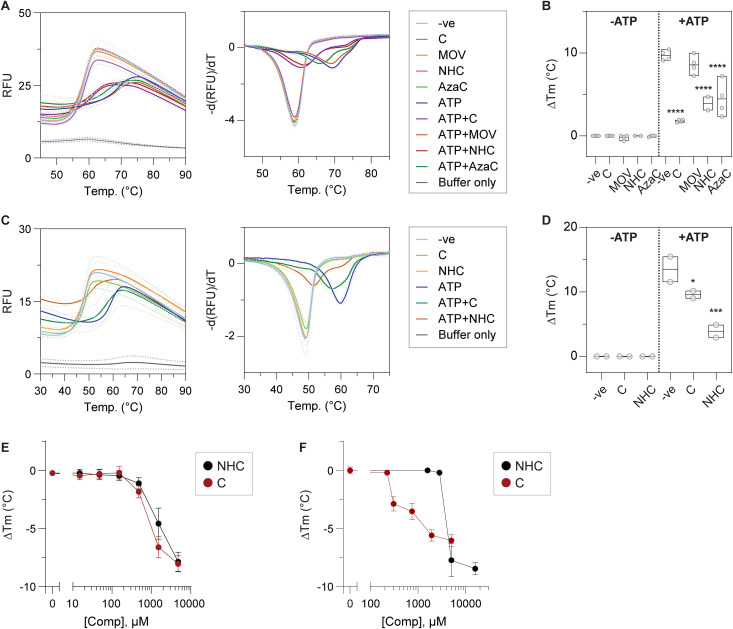
UCK1 and UCK2 preferentially interact with molnupiravir metabolite NHC in their ATP-bound state, similar to other known UCK substrates. **A-B.** Melting profiles of recombinant UCK1 in the presence of potential ligands, determined using differential scanning fluorometry (DSF) assays. Recombinant UCK1 (5 µM) was mixed with 5 mM molnupiravir (MOV), its phosphorylatable metabolite NHC, and known substrates azacitidine (AzaC) or cytidine **(C)**, in the presence or absence of 5 mM ATP. Subsequently, protein thermal stability was examined using DSF. Mean fluorescence signals (solid line) ± SEM (dashed line) of a representative experiment performed in quadruplicate are shown in (A, *left panel*); melting temperatures (Tm) were then determined as the minima of negative derivative of the melting curve (A, *right panel*). In B, mean change of Tm (ΔTm) compared to protein only group of n = 4 independent experiments are shown. Similar to the known substrates of UCK1 cytidine and azacitidine, the phosphorylatable metabolite of molnupiravir NHC, but not molnupiravir, effectively engaged recombinant UCK1 upon the addition of ATP, evidenced by significant Tm changes compared to UCK1 bound to ATP only. Ordinary one-way ANOVA tests (Bonferroni’s multiple comparisons tests) were performed across treatment groups [ΔTm (protein only+ATP) vs. ΔTm (protein only+C), p < 0.0001, t = 12.73, DF = 26; ΔTm (protein only+ATP) vs. ΔTm (protein only+MOV), p = 0.3244, t = 1.815, DF = 26; ΔTm (protein only+ATP) vs. ΔTm (protein only+NHC), p < 0.0001, t = 7.641, DF = 26; ΔTm (protein only+ATP) vs. ΔTm (protein only+AzaC), p < 0.0001, t = 8.469, DF = 26], where asterisks signify statistical significance (****p ≤ 0.0001). **C-D.** Melting profiles of recombinant UCK2 in the presence of potential ligands, determined using DSF assays as described in A-B. Recombinant UCK2 (3.5 µM) was incubated with 5mM compounds alone or in the presence of 5 mM ATP, before protein thermal stabilities were determined using DSF. Similar to UCK1, NHC effectively engaged recombinant UCK2, but only in the presence of ATP. Mean fluorescence signals (solid line) ± SEM (dashed line) of a representative experiment performed in quadruplicate are shown (C, *left panel*); melting temperatures (Tm) were then determined as the minima of negative derivative of the melting curve (C, *right panel*). In D, mean change of Tm (ΔTm) compared to protein only group of n = 2 independent experiments performed in triplicate are shown. Ordinary one-way ANOVA tests (Bonferroni’s multiple comparisons tests) were performed across treatment groups [ΔTm (protein only+ATP) vs. ΔTm (protein only+C), p = 0.048, t = 3.00, DF = 6; ΔTm (protein only+ATP) vs. ΔTm (protein only+NHC), p = 0.0007, t = 7.343, DF = 6], where asterisks signify statistical significance (*p ≤ 0.05, **p ≤ 0.01, ***p ≤ 0.001, ****p ≤ 0.0001). **E-F.** Change of Tm of ATP-bound UCK1 (E) and UCK2 **(F)**, in the presence of increasing concentrations of NHC or C, determined using DSF. Recombinant UCK1 and UCK2 were incubated with 5 mM ATP, alone or together with increasing concentrations of NHC or C, before protein Tm were determined using DSF as described in the previous sections. Change of Tm (ΔTm) was calculated by normalizing to protein + ATP only group. Mean ΔTm ± SEM of n = 4 independent experiments performed in quadruplicate are shown.

To delineate mechanistically the roles of UCK1 and UCK2 in NHC metabolism, we first sought to confirm direct interactions between NHC and recombinant UCK1 and UCK2 using differential scanning fluorimetry (DSF). In the assay, ligand binding to recombinant proteins is interrogated based on the change of the protein thermal stability, as reflected by the shift of protein melting temperature (Tm). Despite high sequence homology, recombinant UCK1 and UCK2 displayed drastically different Tms of approximately 60°C and 50°C, respectively. Nevertheless, both UCK1 and UCK2 were significantly stabilized in the presence of ATP by ∼10 °C, which served as a prerequisite to their interactions with substrates, exemplified by further destabilization with known substrates – cytidine and azacitidine (Fig 2A-D). Meanwhile, no such engagement was observed with the non-substrate thymidine (S2A and S2B Fig), supporting the application of DSF to identify UCK interaction with substrates and cofactors.

Both recombinant UCK1 and UCK2 were able to engage NHC, but only in the presence of ATP, similar to the substrates cytidine and azacitidine (Fig 2A-D). This was subsequently confirmed under a dose-response setup, validating direct interaction between NHC and ATP-bound UCK1/2. Noticeably, with UCK1, NHC demonstrated comparable Tm shifts, suggesting similar binding affinity, as the canonical substrate cytidine, but drastically inferior to cytidine in UCK2 (Fig 2E-F and S2C-S2F Fig). Collectively, these data support that UCK1 and UCK2 dictated NHC cellular response through direct phosphorylation and activation.

### NHC is a substrate of UCK1 and UCK2

**Fig 3:**
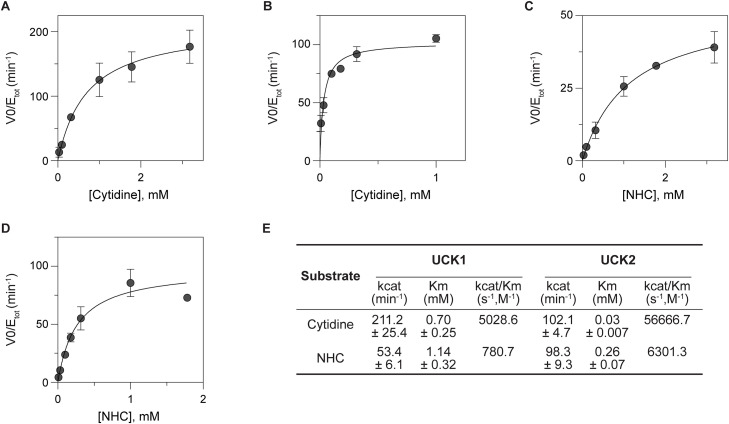
UCK2 is the preferred kinase in phosphorylating molnupiravir metabolite NHC. **A-B.** Saturation curves of UCK1 **(A)** or UCK2 **(B)** -mediated phosphorylation of cytidine, using ADP-Glo-coupled activity assay. **C-D.** Saturation curves of UCK1 **(C)** or UCK2 **(D)** -mediated phosphorylation of NHC, using ADP-Glo-coupled activity assay. In **A-D**, mean initial rates ± SEM of n = 2 independent experiments performed in triplicates are shown. **E.** Kinetic parameters of UCK1 and UCK2-mediated phosphorylation of NHC, in comparison to cytidine, a canonical substrate of UCK1 and UCK2. Kinetic parameters ± SEM were determined via Michaelis-Menten equation (GraphPad Prism) based on the saturation curve shown in **A-D**.

We next directly interrogated NHC as a substrate of UCK1 and UCK2, using an ADP-Glo-coupled kinase activity assay [26]. In the assay, the kinase reaction was coupled to the ADP-Glo detection system (Promega) that converts reaction-generated ADP into measurable luciferase signal, thereby allowing study of the reaction kinetics. Using cytidine as the substrate, the ADP-Glo-coupled kinase activity assay produced kinetic parameters comparable to previous studies for both UCK1 and UCK2 [1], with UCK2 having superior catalytic efficiency of 56666.7 s^-1^ ∙ M^-1^ (Fig 3A-B and S3A-S3D Fig). We were further able to show that NHC was a substrate for both UCK1 and UCK2, with a kcat of 53.4 ± 6.1 min^-1^ and 98.3 ± 9.3 min^-1^, and Km of 1.14 ± 0.32mM and 0.26 ± 0.07 mM, resulting in specificity constants of 780.7 and 6301.3 s^-1^ ∙ M^-1^, respectively (Fig 3C-E and S3E-S3F Fig).

### X-ray crystal structure of human UCK1-NHC-AMPPNP reveals details of substrate binding

**Fig 4:**
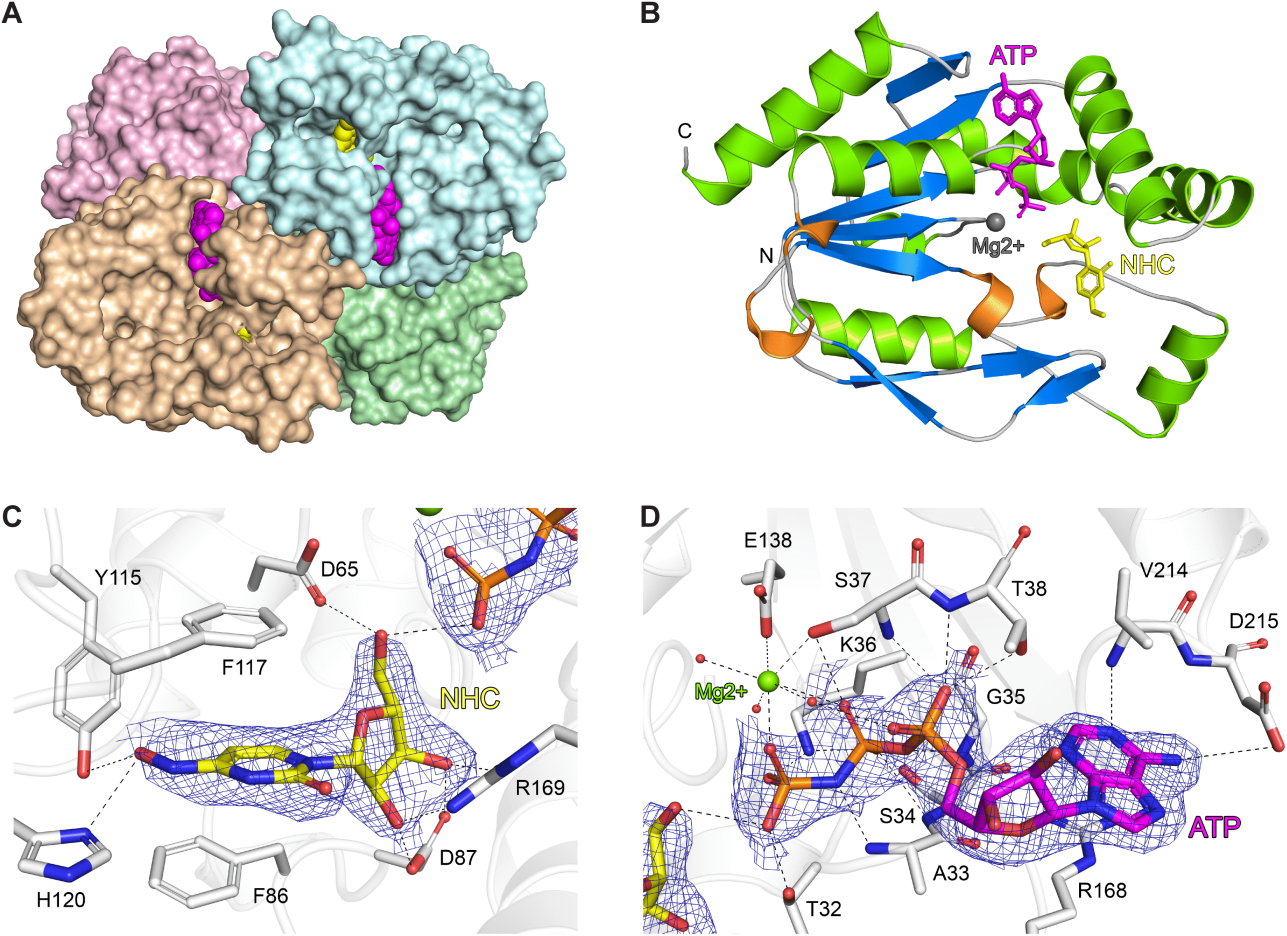
X-ray crystal structure of human UCK1-NHC-AMPPNP. **A.** Surface representation of the UCK1-NHC-AMPPNP tetramer where individual monomers are colored light pink, light cyan, light green and wheat. NHC and AMPPNP are depicted as spheres colored yellow and magenta, respectively. **B.** Cartoon representation of the UCK1-NHC-AMPPNP monomer. Alpha-helices are coloured green, beta-strands are coloured blue, 3_10_-helices are colored orange and loop regions are coloured light grey. The catalytic magnesium ion is shown as a gray sphere. NHC and AMPPNP (labeled as ATP) are shown as sticks colored yellow and magenta, respectively. **C.** Electron density for the NHC binding site. **D.** Electron density for the ATP binding site. The 2*F*_o_-*F*_c_ maps are contoured at 1.0 σ (blue) and the *F*_o_-*F*_c_ maps are contoured at +3.5 σ (green) and -3.5 σ (red). Water molecules are shown as red spheres. Hydrogen bonds are depicted as dashed lines. Figure produced with PyMOL (version 3.0.4, Schrödinger).

We then sought to decipher the structural basis of NHC phosphorylation by UCKs, via X-ray crystallography. We were able to determine the structures of a UCK1 construct (amino acids 21–235), bound with NHC only (UCK1-NHC) or together with the non-hydrolysable ATP analogue AMPPNP (UCK1-NHC-AMPPNP), in space group *P*2_1_ to resolutions of 2.20 and 2.40 Å, respectively (Fig 4) (see S1 Table for data collection and refinement statistics). Consistent with previous studies of UCK2 [27] and size-exclusion chromatography results on recombinant UCK1 (S4A Fig), UCK1 crystallized as a tetramer in the asymmetric unit (Fig 4A and S4B Fig). In the UCK1-NHC-AMPPNP structure, the core exhibited a classical NMP kinase α/β mononucleotide binding fold – a five stranded parallel β-sheet flanked by four α-helices (Fig 4B), with each monomer containing binding sites for NHC (Fig 4C), ATP (Fig 4D) and a single magnesium ion. Within the active site, NHC is supported by π-stacking interactions with F86 and F117, as well as additional H-bond interactions with D65, D87, Y115, H120, R160 and the gamma-phosphate of ATP (Fig 4C). AMPPNP binds in a pocket closer to the surface, and is positioned by H-bonds between the phosphate group and residues T32, A33, G35, K36, S37 and T38, and interactions between the adenine base and amino acids R168, V214 and D215. The single magnesium ion in the structure coordinates with the AMPPNP phosphate group, S37, E138 and three water molecules (Fig 4D).

To understand the structural requirement for ligand interactions with UCK1, we then compared the ligand-bound UCK1 structures with an apo-UCK1 structure (PDB ID: 2JEO). We observed that compared to apo-UCK1, UCK1-NHC and UCK1-NHC-AMPPNP had a slight movement of an α-helix and its connecting loop region, which lines the top of the ligand binding pocket (S4C Fig). Nevertheless, no major structural rearrangements occurred upon ligand binding, as indicated by the low RMSD values of 0.57 Å and 0.61 Å, respectively, following Cα-atom superposition. Upon close inspection of the NHC-binding site, ligand-bound and apo-UCK1 structures differed primarily in the side chain of F117 and the positioning of D65, which are required for engaging NHC via π-stacking and H-bonding respectively (S4D Fig). Notably, this aspartate residue in the UCK2 structure has been proposed to act as a general base in the phosphate transfer reaction [27]. Analysis of the ATP binding site also showed that most residues superimposed well between the structures, with the exceptions being T32, A33, K36 and T38 which shifted slightly in the apo-UCK1 structure, and residue S37 which differed amongst all structures. This is likely due to the role of this serine in magnesium coordination, which is only present in the UCK1-NHC-AMPPNP structure (S4E Fig).

Comparison of UCK1 and UCK2 sequences revealed that the residues coordinating ligand binding are strongly conserved (S4F Fig). Yet, the enzymes displayed different catalytic efficiency (Fig 3). Despite extensive efforts, we were unable to solve a NHC-bound UCK2 structure. Nonetheless, previous work has resolved several crystallography structures of UCK2, in the ligand-free state (PDB ID: 1UFQ), as well as when in complex with its substrate cytidine (PDB ID: 1UEJ), product CMP together with ADP (PDB ID: 1UJ2), or feedback inhibitors CTP (PDB ID: 1UDW) or UTP (PDB ID: 1UEI) [27]. To decipher the structural basis underlying the different activities of UCK1 and UCK2, we then compared the complete substrate-bound UCK1 structure (UCK1-NHC-AMPPNP) with an existing product-bound UCK2 structure (UCK2-CMP-ADP, PDB ID: 1UJ2) [27]. These comparisons also provide insights into structural changes that occur upon phosphate transfer. Interestingly, Cα-atom superposition of UCK1-NHC-AMPPNP and UCK2-CMP-ADP indicates that the monomers are very similar overall as indicated by the low RMSD value of 0.81 Å (S4G Fig), with the ligands occupying the same binding sites and being coordinated by highly conserved amino acids, though different positionings were observed for some residues (S4H-S4I Fig). Meanwhile, UCK1 and UCK2 display differences in sequence identity at the protein tetramerization interface (S4J Fig), as well as for the flexible N-and C-terminal regions. Using Protein Interfaces, Surfaces, and Assemblies (PISA) [28], we next showed that UCK2-CMP-ADP, when compared to UCK1-NHC-AMPPNP, displayed a smaller surface area and significantly larger buried surface area, corresponding to a significantly lower ΔG^int^ and higher ΔG^diss^, collectively indicating tighter subunit association and thereby a more stable complex (S4K Fig).

### UCK2 downregulation hampered the anti-SARS-CoV-2 efficacy of NHC by limiting its bioactivation into NHC-TP

**Fig 5:**
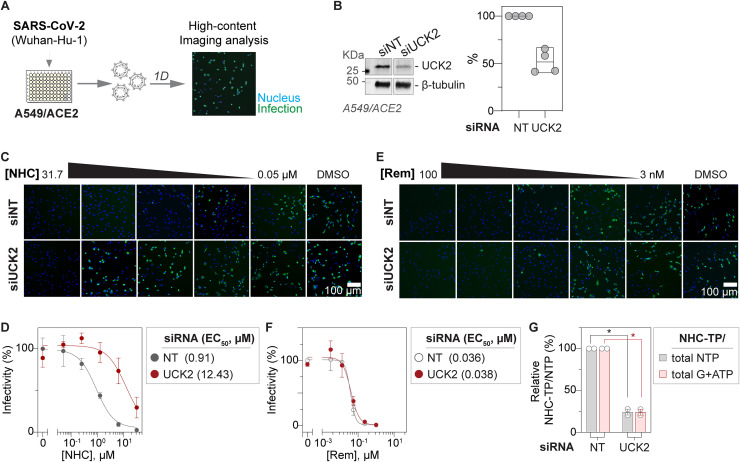
UCK2 expression dictates the anti-SARS-CoV-2 efficacy of NHC. **A.** Schematic representation of the high content imaging-based infectivity assay setup. Lung alveolar basal epithelial A549/ACE2 cells, engineered to overexpress SARS-CoV receptor ACE2, were infected with SARS-CoV-2 at a MOI of 0.07 overnight, before viral infection was assayed via immunofluorescence staining against the viral nucleocapsid protein (NC) and cell nucleus (DAPI). Infectivity was determined as percentage of DAPI/NC double-positive cells out of DAPI single-positive cells, which was further normalized to infectivity level in virus-infected wildtype group and presented as relative infectivity %. **B**. Western blot analysis of A549/ACE2 cell lysates 72 hours post-transfection with UCK2-targeting (siUCK2) or control (siNT) siRNAs. *Left panel*: Representative Western blot image. *Right panel*: densitometry analysis of n = 4 independent experiments, where protein signals were normalized to β-tubulin and relative to that of siNT-treated cells. **C-F.** siRNA-mediated downregulation of UCK2 in A549/ACE2 cells antagonized the anti-SARS-CoV-2 efficacy of NHC **(C-D)**, but not another antiviral nucleoside analogue remdesivir (Rem, **E-F**). At 72 hours post-transfection with UCK2-targeting (siUCK2) or control (siNT) siRNAs, A549/ACE2 cells were re-seeded and infected with SARS-CoV-2 overnight in the presence of antivirals or the drug diluent control DMSO, before viral infectivity was determined using the high content imaging-based infectivity assay as depicted in **A.** In C and E, representative immunofluorescence staining images of infected A549/ACE2 cells, scale bars represent 100 μm; in D and F, mean relative infectivity % ± SEM of n = 3 independent experiments performed in duplicate are shown. **G.** UCK2 knockdown antagonized NHC through limiting the intracellular levels of its tri-phosphorylated active metabolite NHC-TP. A549/ACE2 cells transfected with UCK2-targeting (siUCK2) or control (siNT) siRNAs were treated with 100 µM NHC for 6 hours before cell lysates were harvested for the measurement of intracellular NHC-TP levels. The NHC-TP levels were normalized to total NTP (NHC-TP/total NTP) or total purine-TP (NHC-TP/total NTP G + ATP) levels. Mean NHC-TP/total NTP and NHC-TP/total NTP G + ATP, relative to siNT group, ± SEM of n = 2 independent experiments are shown, together with individual experiment values. Student’s t-tests were performed across treatment groups – for relative NHC-TP/total NTP [siNT vs. siUCK2, p = 0.03, t = 21.5, df = 1]; for relative NHC-TP/total G + ATP [siNT vs. siUCK2, p = 0.03, t = 21.6, df = 1], where asterisk signifies statistical significance (*p ≤ 0.05).

As we have defined the catalysis of NHC by UCKs both kinetically and structurally, we next examined if the *in vitro* kinase activity of UCKs could be pharmacologically relevant for NHC efficacy in cells. For this, we utilized a previously established high-throughput-compatible infectivity assay setup [29] to interrogate the antiviral activity of NHC against SARS-CoV-2, its clinical indication. Specifically, A549/ACE2 cells, a coronavirus susceptible lung basal epithelial cell line overexpressing coronavirus receptor ACE2, were infected with a patient-derived SARS-CoV-2 Wuhan-Hu-1 strain (Pango lineage B; GenBank: MT093571). Viral infectivity was subsequently determined based on the percentage of infected cells as indicated by positive immunofluorescent staining of SARS-CoV-2 nucleocapsid protein (Fig 5Aand S5A-S5B Fig). The platform utilized high-content imaging analysis and automated CellProfiler pipeline for speedy image acquisition and analysis, respectively, thereby accommodating over 3,000 cells per condition and drug dose-response matrix setup. Overall, the assay setup greatly improved experiment throughput and data reproducibility compared to traditional immunofluorescence-based viral quantification methods, e.g., focus-forming assay [30].

To delineate the role of UCK2 in the antiviral efficacy of NHC, we next conducted the SARS-CoV-2 infectivity assay using A549/ACE2 cells transfected with a pool of four UCK2-specific siRNAs (siUCK2) or non-targeting control siRNA (siNT), done in the presence or absence of NHC or another SARS-CoV-2 drug, remdesivir. Both being NA drugs, NHC and remdesivir share a common antiviral mechanism, i.e., targeting viral RNA polymerase for misincorporation into the viral genome upon tri-phosphorylation. Nevertheless, remdesivir is a purine analogue and requires adenylate kinase AK2 for bioactivation [31], thereby serving as the control antiviral drug to decipher potential roles of UCK on NHC efficacy - via the bioactivation versus downstream pathways/targets of NHC. In A549/ACE2 cells with endogenous UCK2 levels, NHC or remdesivir demonstrated micromolar and high nanomolar antiviral EC_50_ values, respectively, agreeing with previous studies [32–34] (Fig 5B-F and S5A-S5B). Upon UCK2 knockdown (KD) via siUCK2 transfection, antiviral efficacy of NHC was significantly hampered as exemplified by a drastic increase of the antiviral EC_50_ value by over 10X, which was not observed for the control drug remdesivir (Fig 5C-F). Of note, during the 24h-course of the infectivity assay, UCK2 KD alone or in combination with either antiviral, had no adverse effect on the host cell proliferation/viability (S5C-S5E Fig).

These data strongly suggest that UCK2 KD antagonized NHC by hampering its bioactivation, which was further supported by determining the intracellular pool of NHC-TP, the antiviral metabolite of NHC. A549/ACE2 cells were transfected with siNT/UCK2 siRNA, and one day post-transfection, cells were treated with NHC followed by intracellular nucleotide extraction and measurement via isocratic reverse-phase HPLC. Whilst endogenous NTP levels were minimally affected, UCK2 KD significantly reduced the intracellular pool of NHC-TP by 75% (Fig 5G and S5F-S5H Fig), directly highlighting the pivotal role of UCK2 in the bioactivation and thereafter antiviral efficacy of NHC.

### UCK is a viable target for tailoring antiviral efficacy of NHC

**Fig 6:**
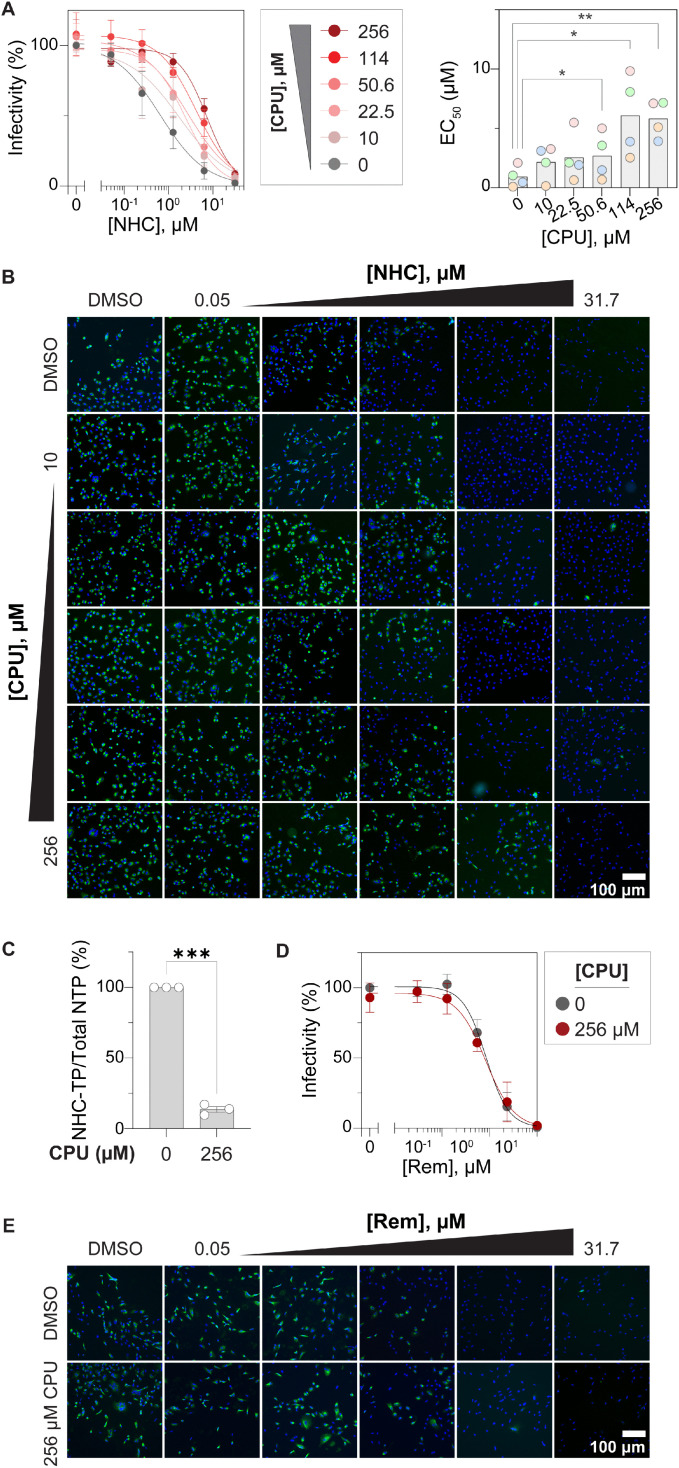
UCK inhibition antagonizes the anti-SARS-CoV-2 efficacy of NHC. **A-B.** CPU-mediated UCK inhibition in A549/ACE2 cells dose-dependently antagonized the anti-SARS-CoV-2 efficacy of NHC. A549/ACE2 cells were infected with SARS-CoV-2 overnight in the presence of dose-response matrix composed of the antivirals/DMSO and CPU/DMSO, before viral infectivity was determined using the high content imaging-based infectivity assay as depicted in Fig 5 **A.** In A, left panel, mean relative infectivity % ± SEM of n = 4 independent experiments performed in duplicate are shown; right panel, mean antiviral EC_50_ of NHC with increasing concentration of CPU are further shown together with color-coded individual experiment values. Paired t-tests were performed across treatment groups – 0 µM CPU vs. 10 µM CPU, p = 0.10; 0 µM CPU vs. 22.5 µM CPU, p = 0.08; 0 µM CPU vs. 50.6 µM CPU, p = 0.05; 0 µM CPU vs. 114 µM CPU, p = 0.03; 0 µM CPU vs. 256 µM CPU, p = 0.003; where asterisk signifies statistical significance (*p ≤ 0.05, **p ≤ 0.01, ***p ≤ 0.001). In B, representative immunofluorescence staining images of infected A549/ACE2 cells, scale bars represent 100 μm. **C.** UCK2 inhibition by CPU significantly hampered the accumulation of intracellular NHC-TP. A549/ACE2 cells were treated with DMSO or 256 µM CPU for 1 hour before cells were further treated with 100 µM NHC for another 5 hours. Cell lysates were subsequently harvested, and the intracellular nucleotide levels were determined. The NHC-TP levels were normalized to total NTP levels and then to DMSO control samples. Mean of the resulting relative NHC-TP levels ± SEM of n = 3 independent experiments are shown. Paired t-test was performed across treatment groups [Relative NHC-TP (0 µM CPU) vs. Relative NHC-TP (256 µM CPU), p = 0.0006, t = 40.51, df = 2], where asterisks signify statistical significance (***p ≤ 0.001). **D-E.** CPU-mediated UCK inhibition in A549/ACE2 cells did not antagonize remdesivir. Experiment was carried out as described in A-B. In D, mean relative infectivity % ± SEM of n = 3 independent experiments performed in duplicate are shown. In E, representative immunofluorescence staining images of infected A549/ACE2 cells, scale bars represent 100 μm.

Given the critical role of UCK in dictating the therapeutic efficacy of NHC/molnupiravir, we next sought to investigate its potential as a pharmacological target for tailoring the drug response. We first investigated if the antiviral efficacy of NHC could be modulated via regulating the catalytic activity of UCK, by employing the cell-active UCK inhibitor CPU. Specifically, A549/ACE2 cells of wildtype UCK expression were infected with SARS-CoV-2 in the presence of increasing concentrations of CPU in combination with NHC or the control antiviral remdesivir, before viral infectivity was determined using the immunofluorescence staining-based infectivity assay. Without inhibiting host cell proliferation/viability (S6A-S6C Fig) or SARS-CoV-2 infection alone (S6D-S6E Fig), UCK inhibitor CPU significantly antagonized the anti-SARS-CoV2 efficacy of NHC in a dose-dependent manner, elevating the antiviral EC_50_ value by up to 6.5X fold compared to the DMSO control group (Fig 6A-B and S6F Fig). Concurrently, direct measurement of the intracellular NTP species revealed an 80% reduction of NHC-TP pool, upon the addition of CPU (Fig 6C and S6G-S6H Fig). Meanwhile, no antagonism was observed for the control antiviral remdesivir (Fig 6D-E and S6I Fig).

**Fig 7:**
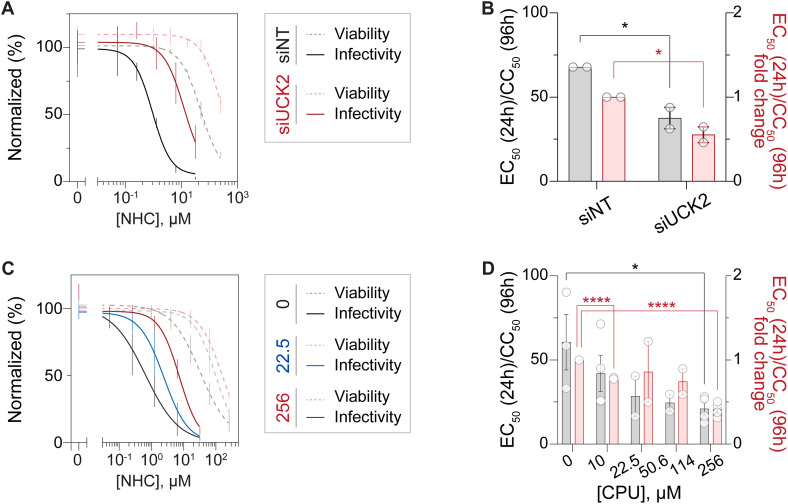
UCK is critical for an optimal selectivity of NHC. **A-B.** Depletion of UCK2 significantly reduced the selectivity (EC_50_ (24h)/CC_50_ (96h)) of NHC by 50%. A549/ACE2 cells transfected with UCK2-targeting (siUCK2) or control (siNT) siRNAs were treated with NHC for 4 days before cell viability was determined using resazurin reduction assay. Alternatively, cells were infected with SARS-CoV-2 overnight in the presence of NHC before viral infectivity was determined using high content imaging-based infectivity assay. Mean viability/infectivity relative to DMSO group ± SEM of n = 2 independent experiments are shown in A, and were further curve fitted using a nonlinear curve fitting model with variable slope (GraphPad Prism) to generate the antiviral EC_50_ and growth inhibitory CC_50_ values of NHC. EC_50_ (24h)/CC_50_ (96h) was then used to indicate drug selectivity. In B, mean EC_50_ (24h)/CC_50_ (96h) ± SEM, and mean fold change of EC_50_ (24h)/CC_50_ (96h) ± SEM are shown. Student’s t-tests were performed across treatment groups [EC_50_ (24h)/CC_50_ (96h) (siNT) vs. EC_50_ (24h)/CC_50_ (96h) (siUCK2), p = 0.041; fold change (siNT) vs. fold change (siUCK2), p = 0.041)], where asterisks signify statistical significance (*p ≤ 0.05). **C-D.** Inhibition of UCK activity by CPU dose-dependently reduced the selectivity (EC_50_ (24h)/CC_50_ (96h)) of NHC, by up to over 50%. A549/ACE2 cells were treated with a dose-response matrix of CPU and NHC for 4 days before cell viability was determined using resazurin reduction assay, or alternatively, cells were infected with SARS-CoV-2 overnight before viral infectivity was determined. Mean viability/infectivity relative to DMSO group ± SEM of n = 2-4 independent experiments are shown in C, and were further curve fitted using a nonlinear curve fitting model with variable slope (GraphPad Prism) to generate the antiviral EC_50_ and growth inhibitory CC_50_ values of NHC. EC_50_ (24h)/CC_50_ (96h) was then used to indicate drug selectivity. In D, EC_50_ (24h)/CC_50_ (96h) ± SEM, and mean fold change ± SEM are shown. Student’s t-tests were performed across treatment groups [EC_50_ (24h)/CC_50_ (96h) (0 µM CPU) vs. EC_50_ (24h)/CC_50_ (96h) (10 µM CPU), p = 0.368; EC_50_ (24h)/CC_50_ (96h) (0 µM CPU) vs. EC_50_ (24h)/CC_50_ (96h) (22.5 µM CPU), p = 0.259; EC_50_ (24h)/CC_50_ (96h) (0 µM CPU) vs. EC_50_ (24h)/CC_50_ (96h) (50.6 µM CPU), p = 0.195; EC_50_ (24h)/CC_50_ (96h) (0 µM CPU) vs. EC_50_ (24h)/CC_50_ (96h) (256 µM CPU), p = 0.042; fold change (0 µM CPU) vs. fold change (10 µM CPU), p = 1.04E-7; fold change (0 µM CPU) vs. fold change (22.5 µM CPU), p = 0.636; fold change (0 µM CPU) vs. fold change (50.6 µM CPU), p = 0.107; fold change (0 µM CPU) vs. fold change (256 µM CPU), p = 8.14E-5], where asterisks signify statistical significance (*p ≤ 0.05, ****p ≤ 0.0001).

A high therapeutic selectivity, i.e., potent antiviral activity with minimal cellular toxicity, is critical for successful clinical application of antiviral drugs. It is determined by the ratio of drug required for 50% cell toxicity (CC_50_) versus 50% viral inhibition (EC_50_), often assayed concurrently. Next, we set out to interrogate the role(s) of UCKs on the selectivity of NHC. Previous studies have suggested that NHC causes cellular stress and potentially cytotoxicity via cumulative DNA damage over multiple replication cycles [16–18]. Agreeing with these data, here we also observed that NHC, whilst induced minimal cytotoxicity with short term treatment concurrent to viral infectivity assay (i.e., 24h) (S5E Fig), prolonged 4-day treatment led to dose-dependent inhibition of the proliferations of multiple cell lines (Fig 1), accompanied by aberrant cell cycle profile, i.e., collapse of replicating S-phase concurrent to G1 arrest (S7A-S7B Fig). To address the divergent antiviral and cytotoxic dynamics of NHC, here NHC’s selectivity is inferred by comparing antiviral EC_50_ determined with 24h treatment, with cytotoxic CC_50_ with prolonged, 96h treatment, i.e., *EC*_*50*_ *(24h)/CC*_*50*_ *(96h)*. Critically, in A549/ACE2 cells, both UCK expression downregulation via siUCK2 (Fig 7A-B), as well as UCK activity inhibition via CPU (Fig 7C-D and S7C Fig), significantly reduced EC_50_ (24h)/CC_50_ (96h) of NHC by up to 2X fold, overall highlighting that UCK is a viable and critical target for achieving optimal antiviral efficacy, and furthermore therapeutic window of the anti-SARS-CoV-2 drug molnupiravir.

## Discussion

Molnupiravir is a broad-spectrum NA antiviral drug against several pathologically important RNA viruses and has been used clinically to treat SARS-CoV-2 infection. Whilst its antiviral mechanism-of-action has been thoroughly studied and deciphered, pharmacogenetic host factors that regulate its therapeutic responses – efficacy as well as selectivity – have not been mechanistically defined and characterized. Here, we identified that host pyrimidine salvage enzymes UCK1 and UCK2 catalyze the first phosphorylation and bioactivation step for NHC – the active compound of molnupiravir – and thereby dictate its antiviral efficacy and more critically, therapeutic window.

In target engagement and enzyme kinetic studies using recombinant enzymes, UCK1 and UCK2 were able to directly bind to NHC and catalyze its phosphorylation (Figs 2-3). Subsequently, using a SARS-CoV-2 infectivity model, downregulation of host cell UCK2 expression via siRNA transfection led to NHC resistance (Fig 5) exemplified by a drastic reduction of intracellular NHC-TP – the active metabolite of molnupiravir – and consequently a 10X increase of the drug antiviral EC_50_. Similar to our data with NHC, UCKs have been demonstrated to phosphorylate and thereby activate other NA drugs, such as azacitidine [1]. The principal mechanism of azacitidine resistance has been attributed to loss-of-activity mutations of UCK2 and loss-of-expression of UCK1, in leukemic cell line models [7,8,35] and patients [6], respectively. This underscores that UCKs could be biomarkers informing treatment outcome for NA drugs, and our data extends this to molnupiravir/NHC. Whilst few catalytically dead UCK2 variants have been identified in leukemic cell lines [8], no studies have been conducted to recapitulate these findings in patient material, or to identify such variants for UCK1 [6]. To fully decipher the potential of UCK1 and UCK2 as clinically applicable biomarkers for NA therapy including molnupiravir/NHC, we therefore envision that patient variant profiling of UCKs and subsequent correlation to drug response should be the focus of future studies.

As UCKs phosphorylated NHC and further controlled its antiviral efficacy, we next investigated if they are pharmacologically tractable targets for tailoring NHC response. To that end, we employed a pan-UCK inhibitor CPU in the SARS-CoV-2 infectivity assay, and observed that CPU dose-dependently antagonized the antiviral efficacy of NHC (Fig 6). More interestingly, with both CPU treatment and UCK2 knockdown, the hampered efficacy is accompanied by a narrowed selectivity (EC_50_ (24h)/CC_50_ (96h)) of NHC, collectively suggesting that elevated UCK expression/activity promotes optimal drug efficacy and therapeutic window (Fig 7). NHC, upon being converted to NHC-TP, is used by the viral polymerase in place of CTP and is then ambiguously matched with GTP or ATP in the subsequent replication cycles, leading to a lethal mutation burden in the viral genome and generation of infection-defective progeny virions [15]. Similarly, a recent study showed that NHC could also induce these mutations in cellular genome, though not of human origin, but mouse lymphoma and myeloid cell lines [17]. The study further implicated UCK2 as the principal driver for such mutations [17]. Nevertheless, the study did not mechanistically characterize the phenotypic consequences of these mutations, in cell cycle progression, cell viability, and/or cell proliferation, making the role of UCK2 in NHC-induced cytotoxicity inconclusive. Here in our study, when using CPU at concentrations where antagonism of antiviral efficacy is consistently observed (e.g., 50–256 µM), minimum rescue of the NHC-induced cytotoxicity was achieved (S7C Fig), supporting that NHC can induce cytotoxicity through additional pathway(s)/drug metabolites aside from those activated via UCKs, e.g., oxidative DNA damage induced by hydroxylamine, a NHC metabolite generated via cytidine deaminase [18,19]. Of note, due to the delayed cytotoxic response of NHC compared to its antiviral activity, the drug selectivity in this study is determined by comparing CC_50_ values at 96h versus EC_50_ values at the earlier, 24h time-point. Future mechanistic studies on NHC-induced cellular responses would not only reveal holistically its cytotoxic mechanisms, but also allow more accurate, timepoint-matched measurement of NHC’s selectivity index. We also envision that future study should focus on systematic profiling of host metabolic factors governing NHC/molnupiravir efficacy and selectivity, which could potentially provide additional targets, alone or in combination with UCKs, for achieving optimal drug responses against its clinical indication SARS-CoV-2, as well as other pathologically important RNA viruses.

UCKs have two isoforms of different catalytic efficiencies, with UCK2 often displaying superior activities compared to UCK1, for the canonical substrates cytidine and uridine as well as NA drugs [1]. Here, in kinetic studies using recombinant UCKs, UCK2 showed markedly higher activities towards NHC as compared to UCK1, displaying a 9-fold higher catalytic efficiency (Fig 3). To gain insights into the basis of this difference, we determined the co-crystal structure UCK1-NHC-AMPPNP, the first complete substrate bound structure of either UCK1 or UCK2. Comparing UCK1-NHC-AMPPNP to a complete product bound structure of UCK2, specifically UCK2-CMP-ADP, the substrate (NHC)/product (CMP) pockets and the ATP/ADP-binding sites showed strong structural conservation. Interestingly, comparative PISA analysis on the two structures to assess quaternary structure stability showed that UCK2-CMP-ADP is significantly more stable compared to UCK1-NHC-AMPPNP, suggesting tighter subunit association in UCK2 and thereby greater structural rigidity. Meanwhile, the less stable tetramerization interface of the UCK1 structure may reflect higher structural flexibility, leading to more dynamic regulation of enzymatic activities.

Notably, UCK2 expression is often limited to embryo and placenta and only upregulated in tissues upon malignant transformation, whilst UCK1 is more ubiquitously expressed, relevant to the multiorgan tropism of coronavirus infection [36]. Our kinetic studies revealed that, compared to UCK1, UCK2 had a 5 times lower Km – 0.26 ± 0.07 mM (UCK2) vs. 1.14 ± 0.32 mM (UCK1), and only a two-fold higher kcat – 98.3 ± 9.3 min^-1^ (UCK2) vs. 53.4 ± 6.1 min^-1^ (UCK1), suggesting its higher catalytic efficiency towards NHC was mainly attributed to the higher substrate binding affinity. With sustained and higher intracellular accumulation of NHC and metabolites compared to the drug level in plasma [37,38], the inferior affinity of UCK1 towards NHC could potentially be overcome *in vivo*, making UCK1 equally relevant to NHC bioactivation. Established cell lines however are often of cancerous/transformed origins, and thus express high levels of UCK2 [39] and potentially have metabolic re-wiring [2], collectively confounding the role(s) of UCK1. Hence, to fully evaluate and decipher the involvement of UCK1 in controlling NHC/molnupiravir efficacy, we envision future studies employing patient materials/data are warranted.

In summary, our study establishes the pivotal role of UCKs in dictating molnupiravir therapeutic efficacy and highlights these enzymes as viable targets for tailoring its response, providing further incentive to develop UCK probes – inhibitory and activating – which is currently an understudied area of research.

## Methods and materials

### Resource availability

#### Lead contact.

Further information and requests for resources and reagents should be directed to and will be fulfilled by the lead contact, Si Min Zhang (simin.zhang@scilifelab.se).

#### Materials availability.

Materials and compounds generated in this study are available from the corresponding authors on reasonable request.

### Experimental model and study participant details

#### Reagents.

*Antibodies* – The antibody against SARS-CoV-2 nucleocapsid protein (mouse, cat. No. MA1–7404) was purchased from Invitrogen. Antibodies against UCK1 (rabbit, cat. No. 12271–1-AP) and UCK2 (rabbit, cat. No. 10511–1-AP) were purchased from Proteintech. Antibody against β-actin (rabbit, cat. no. ab6046) was purchased from Abcam. Donkey anti-rabbit IRDye 800CW (cat. No. 926–32213) was purchased from Li-Cor. Alexa Fluor 488-conjugated goat anti-mouse IgG (H + L) cross-adsorbed secondary antibody (cat. No. A21202) was purchased from Invitrogen.

*Chemicals* – Cyclopentenyl uracil (CPU, cat. No. SML2972–5MG), cytarabine (cat. No. C1768), gemcitabine (cat no. G6423), 5’-azacitidine (cat. No. A2385), cytidine (cat. No. C4654-5G), thymidine (cat. No. T9250), and ATP (cat. No. A2383-1G) were purchased from Sigma-Aldrich; cyclopentenyl cytosine (CPC, cat. No. NSC 375575) was acquired from NCI; 3’-deazauridine (DAU, cat. No. ND16524) was purchased from Carbosynth; molnupiravir (cat. No. HY-135853), NHC (cat. No. HY-125033), and NHC-TP (cat. No. HY-135867) were purchased from MedChemExpress; idoxuridine was purchased from Fluka Biochemika; decitabine (cat. No. QB-5169) was purchased from Combi-Blocks; and floxuridine was purchased from Ega-Chemie. ATP was dissolved in ddH_2_O at 50 mM and stored at -20 °C, and other chemicals were dissolved in DMSO and stored at 4 °C.

*Recombinant protein* – For differential scanning fluorometry and enzymatic activity studies, recombinant UCK1 with a N-terminal His tag was expressed and purified by Protein Science Core Facility (PSF), Karolinska Institutet, Sweden; and recombinant UCK2 with a C-terminal His tag (cat. No. NBP1-50888-0.1mg) was obtained from Novus Biologicals. The expression plasmid pET-15b-UCK1 encoding a human UCK1 construct with N-terminal His-tag was kindly provided by Professor Anna Karlsson (Karolinska Institutet, Sweden) [1]. Briefly, the expression plasmid pET-15b-UCK1 was transformed into *E. coli* BL21 (DE3) T1R pRARE2 cells. The cells were cultivated in Terrific Broth (TB) medium and protein expression was induced by 0.5mM isopropyl-β-D-1-thiogalactopyranoside (IPTG). Cells were then harvested by centrifugation and resuspended in IMAC lysis buffer supplemented with cOmplete Mini, EDTA-free protease inhibitor (Roche), and then stored at -80 °C. During protein purification, cell pellets were thawed with benzonase (2.5 U/mL, Merck-Millipore) and then disrupted by pulsed sonication (4s/4s 4 min, 80% amplitude), before lysates were subjected to centrifugation (20 min at 49000 × g) followed by filtering through 0.45 μm filters. Protein was then purified from the clarified lysates firstly on a HisTrap column (GE Healthcare), using buffer A (20 mM HEPES, 500 mM NaCl, 10% glycerol, 10 mM imidazole, 0.5 mM TCEP, pH 7.5) as starting buffer and an imidazole gradient (10–500 mM) in buffer A as the elution buffer. Protein was then further purified using a Gel Filtration Column, HiLoad 16/60 Superdex 200 (GE Healthcare) in 20 mM HEPES, 300 mM NaCl, 10% glycerol, 0.5 mM TCEP, pH 7.5. Protein-containing fractions were confirmed by SDS-PAGE, pooled in storage buffer (20 mM HEPES, 300 mM NaCl, 10% glycerol, 2 mM TCEP, pH 7.5), aliquoted and stored at -80°C. Protein purities were confirmed using SDS-PAGE and Coomassie staining (see S8 Fig), and concentrations were determined by NanoDrop (Thermo Fisher Scientific) A280 measurement.

For X-ray crystallography studies, a human *uck1* cDNA construct (amino acids 21–235) with an N-terminal His tag, and optimized for expression in *E. coli,* was subcloned into a pET24a(+) vector (GenScript). Protein was expressed in *E. coli* BL21 (DE3) cells overnight at 18 °C using a LEX Bioreactor. The cells were harvested and resuspended in lysis buffer (100 mM HEPES pH 7.5, 500 mM NaCl, 10 mM imidazole, 10% glycerol, 0.5 mM TCEP) and then lysed via sonication. The lysate was clarified by ultracentrifugation for 1 hour at 40,000 rpm. Protein was purified to homogeneity using immobilized metal affinity chromatography (IMAC) followed by size-exclusion chromatography (SEC) using a HiLoad 16/600 Superdex 200 pg column (Cytiva) equilibrated with SEC buffer (20 mM HEPES pH 7.5, 300 mM NaCl, 10% glycerol, 0.5 mM TCEP). Purified protein (see S9 Fig) was concentrated to 12.8 mg/mL, flash frozen in liquid nitrogen and then stored at −80 °C.

#### Biological Resources.

*Cell lines –* LCL-889 cells were obtained by immortalizing healthy donor B cells, as described previously [40]. HL-60 (cat. No. CCL-240), THP-1 (cat. No. TIB-202), MV-4–11 (cat. No. CRL-9591), KG-1a (cat. No. CCL-246.1), MOLT-4 (cat. No. CRL-1582), CCRF-CEM (cat. No. CCL-119), and Daudi (cat. No. CCL-213) were acquired from ATCC. Ramos, BL-41, Raji, MOLT-16, Wil2.NS, and Wil2.NS.6TG cells were acquired from DSMZ. PL-21 was kindly gifted by Dr. Sören Lehmann (Karolinska Institutet, Sweden); Daoy was kindly gifted by Dr. Fredrik Johansson Swartling (Uppsala Universitet, Sweden); UW228-3 was kindly gifted by Dr. John Inge Johnsen (Karolinska Institutet, Sweden); U-343 MG and U-251 MG were kindly gifted by Dr. Lars Braeutigam (Karolinska Institutet, Sweden); and LN-229 was kindly gifted by Dr. Francoise Dantzer (University of Strasbourg, France); A549/ACE2 and Vero E6 were kindly gifted by BSL3 Biomedicum Core Facility (Karolinska Institutet, Sweden).

THP-1, HL-60, and KG1a were cultured in IMDM medium (cat. No. 12440061); U-251 MG, LN-229, and Vero E6 were cultured in DMEM medium with high glucose and GlutaMAX (cat. No. 31966047); Daoy and U343 MG were cultured in MEM medium with GlutaMAX (cat. No. 41090036), supplemented with 1mM pyruvate and 1X NEAA (cat. No. 11350912); UW228-3 was cultured in DMEM/F12 medium with Glutamax (cat. No. 10565018); A549/ACE2 was cultured in DMEM/F12 medium with Glutamax supplemented with 1X NEAA; and all the other cell lines were cultured in RPMI 1640 with GlutaMAX (cat. No. 61870044), at 37 °C with 5% CO_2_ in a humidified incubator. All the cell lines were cultured in medium supplemented with 10% heat-inactivated fetal bovine serum (cat. No. 10500064) and penicillin/streptomycin (100 U/mL and 100 µg/mL, respectively, cat. No. 15070063), except for KG-1a and PL-21, which were cultured in 20% fetal bovine serum and penicillin/streptomycin (100 U/mL and 100 µg/mL, respectively). All cell culture reagents were purchased from ThermoFisher Scientific. The cell lines were regularly monitored and tested negative for the presence of mycoplasma using a commercial biochemical test (MycoAlert, Lonza).

*Virus –* The clinical isolate SARS-CoV-2 (Pango lineage B; GenBank: MT093571) was propagated in African green monkey kidney epithelial cells Vero E6 cells. Briefly, viral inoculated cells were cultured in DMEM medium supplemented with 5% fetal bovine serum and penicillin/streptomycin (100 U/mL and 100 µg/mL, respectively) for 72 hours at 37 °C with 5% CO_2_ in a humidified incubator, till cytopathic effect was apparent. Cells were subsequently harvested, and supernatant was recovered via centrifugation at 2000 rpm for 5 minutes. Clarified supernatant was then aliquoted and stored at -80 °C. Viral titre was determined using plaque assay. Vero E6 cells at 80% confluency in a 6-well plate were inoculated with serially diluted virus aliquots in DMEM medium at 37 °C for 1 hour. Subsequently, cells were washed with PBS twice and overlaid with 0.4% carboxymethylcellulose in MEM supplemented with 5% fetal bovine serum and penicillin/streptomycin (100 U/mL and 100 µg/mL, respectively) at 1mL/well, followed by incubation for 48 hours at 37 °C with 5% CO_2_ in a humidified incubator, till plaques were visible. Cells were then fixed with 10% formaldehyde for 1 hour at room temperature, followed by washing with ddH_2_O thrice and incubation with crystal violet for 30 minutes at room temperature. Following washing in ddH_2_O and air-drying, plaques were then counted and used to estimate the viral titre following the formula $Viral\;titer={Avg}_{\#\;plaques}/(Dilution\;factor\;\times Inoculum\;volume)$.

*Bacteria - E. coli* BL21(DE3) was obtained from Invitrogen.

### Method details

#### In silico UCK substrate screening library composition.

In silico substrate screening was performed on a curated library composed of 3323 therapeutics, sourced from SelleckChem (Catalog No. L7200) and pharmacopeia (Catalog No. L1300). Compound information including (1) name, (2) formula, (3) CAS, and (4) coordinates, was extracted and was subsequently used to identify potential substrates for UCKs, based on structural similarity to the canonical substrates of UCKs, i.e., cytidine and uridine.

#### Cell viability assays.

*Resazurin reduction viability assay* – The resazurin reduction viability assay was conducted as described previously [41,42]. Briefly, suspension and adherent cells were seeded into 384-well or 96-well assay plates at 50,000/mL or 25,000/cm^2^, respectively; and compounds of indicated concentrations were added using a D300e Digital Dispenser (Tecan). When applicable, DMSO levels were normalised across the plate at the maximum level of 1%. Suspension cells were treated on the same day of cell seeding and adherent cells were treated one day post-seeding. Cells were then incubated at 37 °C with 5% CO_2_ in a humidified incubator for 1 or 4 days. On the day of assaying, cells were incubated with resazurin sodium salt (10 µg/mL) for 4–6 hours and the reduction of resazurin by viable cells were subsequently assessed by measuring fluorescence intensity at 544/590 nm (Ex/Em) using a HidexSense plate reader (Hidex). Relative cell viabilities were calculated by subtracting signals of medium-only negative control wells, and then normalized to signals of cell-only positive control wells. When applicable, relative cell viabilities were used to generate compound CC_50_ values *via* a nonlinear curve fitting model with variable slope (GraphPad Prism), as well as to calculate drug combination synergy scores using the Bliss model in SynergyFinder (https://synergyfinder.fimm.fi/) [43].

*Cell growth assessment via cell confluency* – Growth of A549/ACE2 cells of indicated treatment was assessed via cell confluency readout module of Tecan Spark Cyto, and cell confluency % was determined by the machine inbuilt analysis module.

#### Western blot.

Cells were harvested by centrifugation at 400 × g for 4 minutes or trypsinization followed by centrifugation, and were subsequently lysed on ice for 30 minutes using RIPA buffer supplemented with cOmplete Mini, EDTA-free protease inhibitor (Roche) and Halt phosphatase inhibitor (Thermo Fisher Scientific). Lysates were then clarified via centrifugation at 12000 × g for 20 minutes, followed by protein concentration determination using Pierce BCA protein assay kit (Thermo Scientific), per manufacturer’s instruction. For Western blot, clarified cell lysates were mixed in β-mercaptoethanol-supplemented Laemmli buffer (Bio-Rad) and then heated at 99°C for 5–10 min. Lysates containing 15–20 µg protein were subject to sodium dodecyl sulfate-polyacrylamide gel electrophoresis (SDS-PAGE) using 4–15% Mini-PROTEAN TGX gels, and proteins were subsequently transferred to nitrocellulose membranes using a Trans-Blot Turbo machine (Bio-Rad). Following blocking with Odyssey Blocking Buffer (LI-COR), membranes were first probed with primary antibodies at RT for 1 h or 4 °C overnight, and then probed with species-appropriate secondary antibodies at RT for 30 min. Between incubations, membranes were washed trice using TBST (Tris-buffered saline, 0.1% Tween 20). Protein bands were visualised using an Odyssey Fc Imaging System (Li-Cor Biosciences), and subsequently analysed using Image Studio Lite (Ver. 5.2, Li-Cor Biosciences). All uncropped western blot images are provided in the source data.

#### Differential scanning fluorometry (DSF).

DSF assay on recombinant UCK was conducted as described previously, with minor modifications [41]. Briefly, in Axygen 384-well PCR microplate, 5 µM recombinant UCK proteins in 20 µL assay buffer (50 mM Tris-HCl (pH = 7.5), 5 mM MgCl_2_, 100 mM KCl, 2 mM DTT) fortified with Sypro Orange (10X, Invitrogen), were mixed with 5 mM ATP, alone or in the presence of compounds of indicated concentrations. The assay mixture was then subject to a 20–85/90 °C temperature gradient for 20 min, with the fluorescence intensities (RFU) measured every second using a LightCycler 480 Instrument II (Roche Life Science). Melting curves and negative derivative (-*d*RFU/*d*T) transformation were subsequently generated, and melting temperatures (Tm) were identified as the local minima of the negative derivative curves, done using the machine inbuilt software LightCycler 480 SW 1.5.1.

#### ADP-Glo-coupled UCK kinase activity assay.

UCK kinase activity was determined by coupling the kinase reaction to ADP-Glo kinase assay (Promega). In 384-well plates, recombinant UCK protein in 5 µL reaction buffer (50 mM Tris-HCl, pH = 7.5, 5 mM MgCl2, 100 mM KCl, 2 mM DTT, 0.5 mg/mL BSA) was mixed with ATP at a final concentration of 50 µM, followed by the addition of substrates at indicated concentrations to initiate the reaction. For enzyme titration, substrate final concentration was 100 µM for both cytidine and NHC; and the final concentration of UCK1 ranged between 0.00625 to 1.6 µM for cytidine and 0.1 to 1.6 µM for NHC, and UCK2 ranged between 0.938 to 15 nM for cytidine and 3.75 to 60 nM for NHC. For kinetic studies, the final concentration of UCK1 was 6.25 nM for cytidine and 41.6 nM for NHC, and UCK2 of 0.9375 nM for cytidine and 3.75 nM for NHC.

Reactions then proceeded up to 100 min, which were terminated with 8–20 min intervals with the addition of 5 μL ADP-Glo reagent (ATP depletion reagent) followed by incubation at room temperature for 1 hour. Subsequently, 10 μL kinase detection reagent was added followed by 1 hour incubation at room temperature in the dark. Subsequently, luminescence was detected on a HidexSense plate reader (Hidex). ADP formation was calculated using an ADP/ATP conversion standard curve, and was further used to estimate the initial reaction rates and subsequently, kinetic parameters, via Michaelis-Menten equation (GraphPad Prism).

#### Protein crystallisation.

Purified recombinant UCK1 (amino acid 21–235) (12.8 mg/mL) was crystallized via sitting drop vapour diffusion at 21 °C in condition C2 of Morpheus Screen: 0.09 M NPS, 0.1 M Buffer System 1 pH 6.5, 50% (v/v) EDO_P8K (Molecular Dimensions). Protein crystals appeared within 1 day and were soaked for 1 hr in a solution consisting of the respective growth condition supplemented with 10 mM NHC and 10 mM of the non-hydrolysable ATP analogue adenosine 5’-(β,γ-imido)triphosphate (AMPPNP) (Sigma-Aldrich). The crystals were then flash frozen in liquid nitrogen without additional cryoprotectant.

#### Data collection, structure determination and refinement.

X-ray diffraction data was collected on the I03 beamline of the Diamond Light Source (Oxford, UK). All datasets were collected at 100 K using single crystals, at a wavelength of 0.976 Å. Data indexing, integration and scaling was performed using autoPROC [44]. The structures were solved via molecular replacement with PHASER [45] using the AlphaFold model of human UCK1 (AF-Q9HA47) as the search model. Several rounds of manual model building and refinement were performed using Coot [46] and REFMAC5 [47] during which waters and ligands were incorporated into the structures. Data processing and refinement statistics are presented in S1 Table. The coordinates and structure factors for UCK1-NHC-AMPPNP and UCK1-NHC were deposited in the PDB under the codes 9SGG and 9SGF, respectively.

#### RNA interference transfection.

Transfections were performed using INTERFERin (Polyplus Transfection) following manufacturer’s instructions. UCK2-targeting siRNA pool (ON-TARGETplus siRNA SMARTpool; L-005077-00-0005, Dharmacon) or control siRNA (AllStars Negative Control, Qiagen) were transfected at 30 nM final concentration.

#### High-content imaging analysis-guided SARS-CoV-2 infectivity assay.

High-content imaging-guided SARS-CoV-2 infectivity assay was performed as previously described, with minor modifications [29]. All infectious SARS-CoV-2-related work was conducted in the high-containment BSL3 Biomedicum Core Facility at Karolinska Institutet. Briefly, A549/ACE2 cells in suspension were infected with SARS-CoV-2 viruses at an MOI (multiplicity of infection) of 0.07 in serum-free DMEM/F12 medium with Glutamax supplemented with 1X NEAA, at 37 °C with 5% CO_2_ in a humidified incubator under gentle shaking. One hour post-infection, virus-containing medium was removed and replaced with complete medium, and when applicable, medium containing indicated compounds or DMSO, followed by overnight incubation at 37 °C with 5% CO_2_ in a humidified incubator. Cells were then washed with PBS twice, fixed in 4% paraformaldehyde for 20 minutes at room temperature, and washed again with PBS twice and stored at 4 °C till immunofluorescence staining.

To detect virus-infected cells, cells were stained with anti-SARS-CoV-2 nucleocapsid (NC) monoclonal antibody (B46F) (1: 120 in 4% fetal bovine serum/PBS) at 4 °C overnight, followed by Alexa488-conjugated anti-mouse secondary antibody (1: 500 in PBS) mixed with 1 µg/ml DAPI at room temperature for 1–2 h. Cells were washed by PBS thrice between each incubation. Finally, images of cells were acquired on an ImageXpress (Molecular Devices) microscope, and SARS-CoV-2 NC and/or DAPI-positive cells were identified and quantified using Cell Profiler software. For each assay condition per experiment, ≥ 3000 cells were analysed. Percentage of infection is calculated as the ratio between number of DAPI/SARS-CoV-2 NC double-positive cells and total number of DAPI-positive cells, which is further normalized to untreated, virus-infected samples. When applicable, infectivity percentages were used to generate compound antiviral EC_50_ values *via* a nonlinear curve fitting model with variable slope (GraphPad Prism).

#### Cell cycle analysis.

Cells in clear-bottomed 96-well plates (BD Falcon) were subsequently fixed with 4% paraformaldehyde in PBS for 20 minutes, permeabilized with 0.1% Triton X-100 in PBS for 30 minutes, blocked with 4% fetal bovine serum for 30 minutes, and finally stained with 1 µg/ml DAPI in PBS for 1 hour. Alternatively, cells were treated with 10 µM EdU for 30 min before cells were washed twice with PBS followed by fixation, permeabilization, and blocking as described above. Azide-alkyne click reaction was then performed to conjugate EdU with Alexa Fluor 647 Azide (Invitrogen) fluorophore using the following reaction mix – 4 mM CuSO4, 6 nM Alexa Fluor 647 Azide, 10 mM ascorbic acid in PBS. Cells were subsequently stained for DAPI in PBS as described above. All the incubations were performed at room temperature, and cells were washed with PBS twice between each incubation. Images of cells were acquired on an ImageXpress (Molecular Devices) microscope, and then analysed (nuclei counting, DAPI/EdU intensity measurements) with CellProfiler (Broad Institute) and data handled in Excel (Microsoft) and plotted in Prism 8 (GraphPad).

#### Intracellular nucleotide pool measurement.

Intracellular nucleotide pool measurement was performed as described previously, with minor modifications [48,49]. Briefly, A549/ACE2 cells at 70–80% confluency were treated with compounds of indicated concentrations or 1.2% DMSO for 6 hours at 37 °C with 5% CO_2_ in a humidified incubator. Cells were then washed with TBS over ice, and then harvested in 700 μl ice-cold TCA solution (15% trichloroacetic acid, 30 mM MgCl_2_) by using a cell scraper, before being snap frozen in liquid nitrogen and then stored at -80ºC till nucleotide extraction and analysis via isocratic reverse-phase HPLC coupled with UV detection, which were performed as described previously [49].

### Quantification and statistical analysis

Statistical analysis was conducted using GraphPad Prism 9 software. Specific statistical test details, including test method, n values (number of independent experiments), number of technical repeats per independent experiment, and dispersion and precision measures, are indicated in the corresponding figure legends. Statistical significance is defined as p < 0.05, unless otherwise stated. Asterisk in figures signifies statistical significance (* for p ≤ 0.05, ** for p ≤ 0.01, *** for p ≤ 0.001, **** for p ≤ 0.0001).

## Supporting information

S1 Fig Supplemental information of UCK inhibition antagonized NHC-induced cell growth inhibition, related to Fig 1. A. UCK inhibitor CPU dose-dependently antagonized the cytotoxic efficacy of NHC in multiple cell lines. Cells were treated with a dose-response matrix of NHC and CPU for 4 days before cell viability was determined using the resazurin reduction assay. Mean cell viability relative to DMSO control group ± SEM of n = 2–3 independent experiments are shown, which were further curve fitted using a nonlinear curve fitting model with variable slope (GraphPad Prism) to generate the cell viability curves. **B-E**. Ponceau red staining images of Western blot membranes shown in Fig 1E, to demonstrate cell lysates containing equal amounts of total proteins were analysed. In B, same membrane later probed for UCK1 and UCK2, generating Western blot image shown in Fig 1E left panel; in C and D, membranes later probed for UCK1 (C) and UCK2 (D), generating Western blot image shown in Fig 1E right panel. Sample identities are shown in E.
(PDF)

S2 Fig Supplemental information of DSF experiments on recombinant UCK proteins, incubated with nucleotides, related to Fig 2. A-B. The non-substrate thymidine did not effectively engage recombinant UCK1, when applied up to 4.8 mM in the DSF assay. Recombinant UCK1 (5 µM) was incubated with indicated concentrations of thymidine or DMSO, in the presence or absence of ATP, before protein thermal stabilities were determined using the DSF assay. In A, *left panel* displays melting curves of a representative experiment performed in quadruplicate, where mean fluorescence signals (solid line) ± SEM (dashed line) are shown; *right panel* displays negative derivative (-dRFU/dT) of the melting curves in the left panel, where mean negative derivative values (solid lines) ± SEM (dashed lines) are shown. Protein melting temperatures (Tm) were determined as the local minima of the negative derivative curves, and changes of Tm compared to ATP-only group are shown as ΔTm in B, where mean ΔTm ± SEM of n = 4 independent experiments performed in triplicate to quadruplicate are shown. **C-F**. Melting profiles of recombinant UCK1 (C-D) and UCK2 (E-F) when incubated with NHC or C. Recombinant UCK proteins were incubated with increasing concentrations of NHC, C or DMSO, in the presence or absence of ATP, before protein thermal stabilities were determined using the DSF assay. *Left panels*, melting curves of a representative experiment performed in quadruplicate, where mean fluorescence signals (solid line) ± SEM (dashed line) are shown; *right panel*, negative derivative (-dRFU/dT) of the melting curves in the left panel, where mean negative derivative values (solid lines) ± SEM (dashed lines) are shown. The curves are supplemental to Fig 2E-F.
(PDF)

S3 Fig Supplemental information of kinetic studies using the ADP-Glo-coupled kinase activity assay, related to Fig 3. A-B. Titration of UCK1 (A) and UCK2 (B) when using cytidine or NHC as the substrate. Recombinant UCK1 and UCK2 of varying concentrations were incubated with 200 µM or 100 µM substrates, respectively. Reactions were allowed to proceed for indicated periods before the addition of ADP-Glo reagent and kinase detection reagent, with incubation of 1 hour between each addition. Luminescence measurement was then done on a Hidex plate reader. Mean luminescence signals ± SEM of independent experiments performed in triplicate are shown. **C-D**. Kinetic studies of phosphorylation of cytidine by recombinant UCK1 (C) and UCK2 (D), supplementary to Fig 3A and 3B, respectively. The reaction was linear under the specified conditions for the duration of the experiment. Mean luminescence ± SEM of a representative experiment performed in triplicate are shown, which were subsequently used to determine V0 and reaction kinetic parameters. **E-F**. Kinetic studies of phosphorylation of NHC by recombinant UCK1 (E) and UCK2 (F), supplementary to Fig 3C and 3D, respectively. The reaction was linear under the specified conditions for the duration of the experiment. Mean luminescence ± SEM of a representative experiment performed in triplicate are shown, which were subsequently used to determine V0 and reaction kinetic parameters.
(PDF)

S4 Fig Supplemental information of X-ray crystallography studies, related to Fig 4. A. Tetramerization of the purified recombinant UCK1 (amino acids 21–235). Left: Chromatogram from Gel filtration chromatography run performed using a Superdex200 increase 10/300 GL column (Cytiva). The single peak (labelled U) is consistent with the expected size of the hUCK1 tetramer. Right: Corresponding SDS-PAGE gel analysis. 20 micrograms of protein was loaded onto the gel. L: PageRuler unstained protein ladder (Thermo Fisher Scientific). U: UCK1 (amino acids 21–235) protein sample. **B.** Multiple views of human UCK1-NHC-AMPPNP tetramer. Surface representation of the UCK1-NHC-AMPPNP tetramer where individual monomers are colored light pink, light cyan, light green and wheat. NHC and ATP are depicted as spheres colored yellow and magenta, respectively. **C**. Cα-atom superpositions of UCK1-NHC (green) with UCK1-NHC-AMPPNP (blue) and apo-UCK1 (white, PDB ID: 2jeo). The magnesium ion from UCK1-NHC-AMPPNP is shown as a gray sphere. NHC and ATP are depicted as sticks colored yellow and magenta, respectively. **D-E.** Comparison of NHC (D) and ATP (E) binding sites in UCK1-NHC and UCK1-NHC-AMPPNP with apo-UCK1. Amino acid side chains are shown as sticks; C atoms are coloured according to the color scheme shown in panel **C**, O atoms colored red, N atoms colored blue, and P atoms colored orange. The magnesium ion from UCK1-NHC-AMPPNP is shown as a gray sphere. NHC and AMPPNP are depicted as sticks colored yellow and magenta, respectively. Hydrogen bonds from the UCK1-NHC-AMPPNP structure are depicted as dashed lines. **F**. Amino acid sequence alignment of human UCK1 (UniProt ID: Q9HA47) and human UCK2 (UniProt ID: Q9BZX2). Identical residues are shaded black, while grey shading indicates amino acids with conserved physicochemical properties. The secondary structure annotation of UCK1-NHC-AMPPNP is shown below the alignment. Amino acids from UCK1-NHC-AMPPNP required for NHC, ATP or magnesium binding are indicated by boxes above the alignment coloured yellow, magenta or dark grey, respectively. **G.** Cα-atom superposition of UCK1-NHC-AMPPNP (blue) with human UCK2-CMP-ADP (white, PDB ID: 1xrj). The magnesium ion in each structure is shown a sphere coloured grey (UCK1-NHC-AMPPNP) or green (UCK2-CMP-ADP). CMP and ADP are depicted as sticks coloured yellow and magenta, respectively. NHC and AMPPNP are shown as light grey sticks. **H-I.** Comparison of NHC/CMP (**H**) and AMPPNP/ADP (**I**) binding sites between UCK1-NHC-AMPPNP and UCK2-CMP-ADP. Amino acid side chains are shown as sticks; C atoms are coloured according to the color scheme shown in panel **G**, O atoms colored red, N atoms colored blue, and P atoms colored orange. Hydrogen bonds from the UCK2-CMP-ADP structure are depicted as dashed lines. NHC and AMPPNP are shown as thin black lines for clarity. At the substrate/product binding site (**H**), CMP occupied the same site in UCK2 as NHC in UCK1, with the coordinating amino acids entirely conserved though adopting different positioning. Specifically, residues D62, Y112, F114 and H117 of UCK2, i.e., D65, Y115, F117, and H120 in UCK1, differed markedly; and residues R174 and R176 in UCK2, i.e., R178 and R176 in UCK1, shifted even more pronouncedly. In **I**, ADP in UCK2 occupied the same pocket as AMPPNP in the UCK1 structure. Despite two coordinating residues differing – S35 and A212 in UCK2 corresponding to T32 and V214 in UCK1, no significant movements were observed, except for a shift in the catalytic magnesium ion and a flip of the adenine base of ADP in the UCK2 structure. This is likely because serine and threonine have conserved physicochemical properties, and it is the mainchain atom of A212 that coordinates the adenine base rather than its sidechain. **J.** Cartoon representations of UCK1-NHC-AMPPNP tetramer, where amino acid differences between UCK1 and UCK2 are highlighted. Red coloring indicates non-conserved differences and orange coloring indicates differences with conserved physicochemical properties. NHC and AMPPNP are colored yellow or magenta, respectively. Magnesium ions are depicted as grey spheres. **K.** PISA analysis of UCK1-NHC-AMPPNP and UCK2-CMP-ADP, performed using the Proteins, Interfaces, Structures and Assemblies (PISA) webserver. In panel **B-E** and **G-J**, figures were produced with PyMOL (version 3.0.4, Schrödinger).
(PDF)

S5 Fig Supplemental information of UCK2 downregulation-induced loss of NHC antiviral efficacy, related to Fig 5. A-B. The high-content imaging-based SARS-CoV-2 infectivity assay produced antiviral efficacies of NHC (A) and Remdesivir (B) that are comparable to past studies. A549/ACE2 cells were infected overnight with SARS-CoV-2 at an MOI of 0.07 in the presence of antivirals or the diluent control DMSO, before cells were fixed and stained for viral nucleocapsid protein (NC) and nuclei using NC-specific antibody and DAPI, respectively. Infectivity was estimated as the ratio of NC/DAPI double-positive cells over DAPI single-positive cells, and further normalized to DMSO-only control group. The resulting relative infectivity (%) data were then curve fitted using a nonlinear curve fitting model with variable slope (GraphPad Prism) to generate the antiviral EC_50_. The estimated infectivity agreed with mean NC intensity in cells stained positive for DAPI, showcasing the robustness of the automated cell profiler pipeline for identifying NC/DAPI double-positive cells. Mean infectivity relative to DMSO-only control group ± SEM, as well as mean NC intensity/cells/well, of a representative experiment performed in duplicate are shown. **C.** UCK2 knockdown did not affect A549/ACE2 cell cycle progression. A549/ACE2 cells were transfected with UCK2-specific (siUCK2) or non-targeting control (siNT) siRNA. Four days post-transfection, cells were fixed and stained for DAPI, followed by high-content imaging acquisition and DAPI signal analysis using CellProfiler. Frequency distribution analysis of DAPI signals were then done using GraphpadPrism. **D.** UCK2 knockdown did not affect A549/ACE2 cell proliferation. A549/ACE2 cells were transfected with UCK2-specific (siUCK2) or non-targeting control (siNT) siRNA. One day post-transfection, cells were re-seeded and allowed to proliferate for four days, before cell growth was determined via the confluence analysis function on a Tecan Spark Cyto imaging cytometer. *Left panel*, mean cell confluence ± SEM of n = 2 independent experiments; right panel, representative cell growth pictures used for confluence analysis. **E.** During the duration of viral infectivity assay, neither NHC (left panel) nor remdesivir (Rem, right panel), significantly affected the viability of siUCK2 or siNT-transfected A549/ACE2 cells. A549/ACE2 cells were transfected with siUCK2 or siNT siRNA. Three days post-transfection, cells were treated overnight with NHC (left panel), remdesivir (right panel), or the diluent control DMSO, at the same concentrations used for viral infectivity assay as shown in Fig 5C-F. Cell viability was subsequently determined using resazurin reduction viability assay and normalized to DMSO-only control group. Mean relative viability ± SEM of n = 2 independent experiments are shown. **F-H.** UCK2 knockdown significantly hampered intracellular NHC-TP pool, with no effect on NTP levels. A549/ACE2 cells were transfected with siUCK2 or siNT siRNA. One day post-transfection, cells were treated with 100 µM NHC before cell lysates were harvested, and intracellular nucleotide pools were measured via HPLC. In F, mean NTP levels per 10^6^ cells ± SEM of n = 2 independent experiments, together with individual experiment values, are shown. In G, mean NHC-TP levels per 10^6^ cells ± SEM of n = 2 independent experiments, together with individual experiment values, are shown. In H, mean NHC-TP levels normalized to total NTP (NHC-TP/total NTP) or purine-TP levels (NHC-TP/total NTP G + ATP) ± SEM of n = 2 independent experiments are shown, together with individual experiment values. In G-H, Student’s t-tests were performed across treatment groups – for NHC-TP levels per 10^6^ cells [siNT vs. siUCK2, p = 0.0137, t = 8, df = 2]; for NHC-TP/total NTP [siNT vs. siUCK2, p = 0.004, t = 15, df = 2]; for NHC-TP/total G + ATP [siNT vs. siUCK2, p = 0.005, t = 13.4, df = 2], where asterisk signifies statistical significance (*p ≤ 0.05, **p ≤ 0.01).
(PDF)

S6 Fig Supplemental information of CPU-induced loss of NHC antiviral efficacy, related to Fig 6. A. UCK inhibition via CPU did not affect A549/ACE2 cell proliferation, shown via confluence analysis. A549/ACE2 cells were seeded overnight and then treated with indicated concentrations of CPU or the diluent control DMSO for four days before cell growth was assessed using the confluence analysis function on a Tecan Spark Cyto imaging cytometer. *Left panel*, mean cell confluence relative to DMSO-only group ± SEM of n = 2 independent experiments; right panel, representative cell growth pictures used for confluence analysis. Dunnett’s multiple comparisons tests were performed across treatment groups [0 µM CPU vs. 22.1 µM CPU, p = 0.9762, q = 0.3173, DF = 4; 0 µM CPU vs. 75 µM CPU, p = 0.7345, q = 0.8690, DF = 4; 0 µM CPU vs. 256 µM CPU, p = 0.7345, q = 0.8690, DF = 4], where ns signifies statistical insignificance (p > 0.05). **B-C**. During the course of the 1-day viral infectivity assay, NHC and/or CPU did not affect cell viability (B) or cell cycle progression (C). A549/ACE2 cells were treated overnight with a concentration matrix of NHC and CPU, at the same concentrations as in viral infectivity assay shown in Fig 6. Following the treatment, in B, cell viabilities were determined using the resazurin reduction viability assay. Relative viability normalized to cells treated with DMSO only ± SEM of n = 2 independent experiments are shown. In C, cell cycle was determined following the treatment with highest concentrations of NHC and/or CPU, where DAPI-stained cells were imaged by high-content imaging followed by DAPI intensity analysis via CellProfiler. *Left panel*, DAPI-intensity histograms with gating strategy of a representative experiment; *right panel*, mean percentages of cells in different cell cycle phases ± SEM of n = 2 independent experiments, together with individual experiment values, are shown. **D-E.** CPU alone did not inhibit SARS-CoV-2 infectivity. A549/ACE2 cells were infected with SARS-CoV-2 overnight with or without CPU, before viral infectivity and nucleocapsid (NC) protein level per cell were determined using the high content imaging-based infectivity assay. CPU was tested at the same concentrations as in Fig 6. In D, *left panel*, mean relative infectivity % ± SEM of n = 4 independent experiments performed in duplicate are shown; *right panel*, representative immunofluorescence staining images of infected A549/ACE2 cells, scale bars represent 100 μm. In E, NC fluorescence signal was measured for each of the DAPI-positive cells, and then averaged to generate mean NC intensity within each treatment group. Mean NC intensity fold change (FC) compared to DMSO group ± SEM of n = 4 independent experiments are shown. **F**. CPU-indued fold changes of NHC antiviral EC_50_ values, determined via high-content imaging-based infectivity assay. This figure is supplementary to Fig 6A. Mean EC_50_ fold changes, relative to DMSO control group, of n = 4 independent experiments are shown, together with color-coded individual experiment values. Paired t-tests were performed across treatment groups – 0 µM CPU vs. 10 µM CPU, p = 0.10; 0 µM CPU vs. 22.5 µM CPU, p = 0.08; 0 µM CPU vs. 50.6 µM CPU, p = 0.05; 0 µM CPU vs. 114 µM CPU, p = 0.03; 0 µM CPU vs. 256 µM CPU, p = 0.003; where asterisk signifies statistical significance (*p ≤ 0.05, **p ≤ 0.01, ***p ≤ 0.001). **G-H.** CPU significantly hampered intracellular NHC-TP pool, with no effect on NTP levels. A549/ACE2 cells were pre-treated with 256 µM CPU or DMSO for an hour before being further treated with 100 µM NHC or DMSO for 6 hours. Cell lysates were then harvested, and intracellular nucleotide pools were measured via HPLC. In G, mean NTP levels per 10^6^ cells ± SEM of n = 3 independent experiments, together with individual experiment values, are shown. In H, mean NHC-TP levels per 10^6^ cells ± SEM of n = 3 independent experiments, together with individual experiment values, are shown. NHC-TP levels were further normalized to total NTP levels and the resulting NHC-TP/total NTP values ± SEM of n = 3 independent experiments, together with individual experiment values, are also shown in H. Ratio paired t-tests were performed across treatment groups – for NHC-TP levels per 10^6^ cells [0 µM CPU vs. 256 µM CPU, p = 0.0025, t = 20.09, df = 2]; for NHC-TP/total NTP [0 µM CPU vs. 256 µM CPU, p = 0.0066, t = 12.23, df = 2], where asterisk signifies statistical significance (**p ≤ 0.01). **I.** During the course of the 1-day viral infectivity assay, remdesivir (Rem) and/or CPU did not affect cell viability. A549/ACE2 cells were treated overnight with Rem and/or CPU, at the same concentrations as in viral infectivity assay shown in Fig 6, which was then followed by resazurin reduction viability assay to determine cell viability. Relative viability normalized to cells treated with DMSO only ± SEM of n = 2 independent experiments are shown.
(PDF)

S7 Fig Supplemental information of CPU-induced reduction of the selectivity of NHC, related to Fig 7. A-B. Four day-treatment with NHC induced S phase collapse and G1 arrest in A549/ACE2 cells. A549/ACE2 cells were treated with increasing concentrations of NHC for four days before cell cycles were analysed via immunofluorescence staining for EdU incorporation and DAPI followed by high-content imaging. In A, representative plots with gating strategy illustrated. In B, percentage of cells in each cell cycle (*left panel*) and mean integrated EdU signal of cells in S phase (*right panel*) of a representative experiment, illustrating that NHC dose-dependently induced collapse of EdU-incorporating S phase cells concurrent to G1 arrest. **C.** Viral infectivity inhibition curves of SARS-CoV-2 infection in A549/ACE2 cells, and the viability curves of A549/ACE2 cells generated using the same dose-response matrix of CPU and NHC. In the viral infectivity assay, A549/ACE2 cells were infected with SARS-CoV-2 in the absence or presence of CPU and/or NHC. One day post-infection, viral infectivity was determined via staining the cells with viral nucleocapsid protein followed by high-content imaging analysis. Mean relative infectivity normalized to the DMSO-only group ± SEM of n = 4 independent experiments performed in duplicate are shown. Meanwhile the effect of NHC on cell viability was determined with the same NHC/CPU matrix but a prolonged four-day drug treatment, followed by resazurin viability reduction assay. Mean viability relative to DMSO group ± SEM of n = 2–4 independent experiments are shown. Both viability and infectivity data were further curve fitted using a nonlinear curve fitting model with variable slope (GraphPad Prism) to generate the antiviral EC_50_ and growth inhibitory CC_50_ values of NHC, which were further used to calculate EC_50_ (24h)/CC_50_ (96h) as shown in Fig 7D.
(PDF)

S8 Fig Supplemental information of recombinant UCK1 protein production and purification. Purified recombinant UCK1 protein (4 µg) was subject to SDS-PAGE followed by Coomassie brilliant blue staining, demonstrating protein purity.
(PDF)

S9 Fig Supplemental information of recombinant UCK1 (amino acids 21–235) protein production and purification. Recombinant UCK1 (amino acids 21–235) fractions post-gel filtration(fraction number indicated in the picture) was analysed by SDS-PAGE followed by Coomassie brilliant blue staining, demonstrating high protein purity. The fractions shown were then pooled for protein X-ray crystallography studies.
(PDF)

S1 Table X-ray crystallography data collection and refinement statistics. (JPG)

S1 Raw Image Un-cropped Western Blot images for experiments presented in Figs 1E and 5B. (PDF)

S1 Data Minimal dataset. (XLSX)

## References

[1] Van Rompay AR, Norda A, Lindén K, Johansson M, Karlsson A. Phosphorylation of uridine and cytidine nucleoside analogs by two human uridine-cytidine kinases. Mol Pharmacol. 2001;59(5):1181–6. doi: 10.1124/mol.59.5.1181 11306702

[2] Fu Y, et al. The metabolic and non-metabolic roles of UCK2 in tumor progression. Frontiers in Oncology. 2022;12. 10.3389/fonc.2022.904887 PMC9163393 35669416

[3] Drake JC, Stoller RG, Chabner BA. Characteristics of the enzyme uridine-cytidine kinase isolated from a cultured human cell line. Biochem Pharmacol. 1977;26(1):64–6. doi: 10.1016/0006-2952(77)90132-0 64250

[4] Van Rompay AR, Johansson M, Karlsson A. Phosphorylation of deoxycytidine analog monophosphates by UMP-CMP kinase: molecular characterization of the human enzyme. Molecular Pharmacology. 1999;56(3):562–9. 10462544 10.1124/mol.56.3.562

[5] Sarkisjan D, Julsing JR, Smid K, de Klerk D, van Kuilenburg ABP, Meinsma R, et al. The cytidine analog fluorocyclopentenylcytosine (RX-3117) is activated by uridine-cytidine kinase 2. PLoS One. 2016;11(9):e0162901. doi: 10.1371/journal.pone.0162901 27612203 PMC5017758

[6] Valencia A, Masala E, Rossi A, Martino A, Sanna A, Buchi F, et al. Expression of nucleoside-metabolizing enzymes in myelodysplastic syndromes and modulation of response to azacitidine. Leukemia. 2014;28(3):621–8. doi: 10.1038/leu.2013.330 24192812 PMC3948159

[7] Gu X, Tohme R, Tomlinson B, Sakre N, Hasipek M, Durkin L, et al. Decitabine- and 5-azacytidine resistance emerges from adaptive responses of the pyrimidine metabolism network. Leukemia. 2021;35(4):1023–36. doi: 10.1038/s41375-020-1003-x 32770088 PMC7867667

[8] Sripayap P, Nagai T, Uesawa M, Kobayashi H, Tsukahara T, Ohmine K, et al. Mechanisms of resistance to azacitidine in human leukemia cell lines. Exp Hematol. 2014;42(4):294-306.e2. doi: 10.1016/j.exphem.2013.12.004 24368162

[9] Wahl A, Gralinski LE, Johnson CE, Yao W, Kovarova M, Dinnon KH, et al. SARS-CoV-2 infection is effectively treated and prevented by EIDD-2801. Nature. 2021;591(7850):451–7. doi: 10.1038/s41586-021-03312-w 33561864 PMC7979515

[10] Ehteshami M, Tao S, Zandi K, Hsiao H-M, Jiang Y, Hammond E, et al. Characterization of β-d-N4-Hydroxycytidine as a Novel Inhibitor of Chikungunya Virus. Antimicrob Agents Chemother. 2017;61(4):e02395-16. doi: 10.1128/AAC.02395-16 28137799 PMC5365705

[11] Stuyver LJ, Whitaker T, McBrayer TR, Hernandez-Santiago BI, Lostia S, Tharnish PM, et al. Ribonucleoside analogue that blocks replication of bovine viral diarrhea and hepatitis C viruses in culture. Antimicrob Agents Chemother. 2003;47(1):244–54. doi: 10.1128/AAC.47.1.244-254.2003 12499198 PMC149013

[12] Yoon J-J, Toots M, Lee S, Lee M-E, Ludeke B, Luczo JM, et al. Orally efficacious broad-spectrum ribonucleoside analog inhibitor of influenza and respiratory syncytial viruses. Antimicrob Agents Chemother. 2018;62(8):e00766-18. doi: 10.1128/AAC.00766-18 29891600 PMC6105843

[13] Reynard O, et al. Identification of a new ribonucleoside inhibitor of Ebola virus replication. Viruses. 2015;7(12):6233–40. 26633464 10.3390/v7122934 PMC4690858

[14] Agostini ML, et al. Small-molecule antiviral β-d-N(4)-hydroxycytidine inhibits a proofreading-intact coronavirus with a high genetic barrier to resistance. J Virol. 2019;93(24). 10.1128/JVI.01348-19 PMC6880162 31578288

[15] Kabinger F, Stiller C, Schmitzová J, Dienemann C, Kokic G, Hillen HS, et al. Mechanism of molnupiravir-induced SARS-CoV-2 mutagenesis. Nat Struct Mol Biol. 2021;28(9):740–6. doi: 10.1038/s41594-021-00651-0 34381216 PMC8437801

[16] Zhou S, et al. β-d-N4-hydroxycytidine inhibits SARS-CoV-2 through lethal mutagenesis but is also mutagenic to mammalian cells. The Journal of Infectious Diseases. 2021;224(3):415–9. 33961695 10.1093/infdis/jiab247 PMC8136050

[17] Xu Z, Flensburg C, Bilardi RA, Majewski IJ. Uridine-cytidine kinase 2 potentiates the mutagenic influence of the antiviral β-d-N4-hydroxycytidine. Nucleic Acids Res. 2023;51(22):12031–42. doi: 10.1093/nar/gkad1002 37953355 PMC10711452

[18] Mori Y, Yogo R, Kobayashi H, Katsuzaki H, Hirao Y, Kato S, et al. Reactive oxygen species-mediated cytotoxic and DNA-damaging mechanism of N4-hydroxycytidine, a metabolite of the COVID-19 therapeutic drug molnupiravir. Free Radic Res. 2025;59(3):205–14. doi: 10.1080/10715762.2025.2469738 39973207

[19] Kobayashi H, Mori Y, Ahmed S, Hirao Y, Kato S, Kawanishi S, et al. Oxidative DNA Damage by N4-hydroxycytidine, a Metabolite of the SARS-CoV-2 Antiviral Molnupiravir. J Infect Dis. 2023;227(9):1068–72. doi: 10.1093/infdis/jiac477 36461940 PMC10132762

[20] Feng JY. Addressing the selectivity and toxicity of antiviral nucleosides. Antivir Chem Chemother. 2018;26:2040206618758524. doi: 10.1177/2040206618758524 29534607 PMC5890540

[21] Lim MI, Moyer JD, Cysyk RI, Marquez VE. Cyclopentenyluridine and cyclopentenylcytidine analogues as inhibitors of uridine-cytidine kinase. J Med Chem. 1984;27(12):1536–8. doi: 10.1021/jm00378a002 6094806

[22] Karle JM, Cysyk RL. Regulation of pyrimidine biosynthesis in cultured L1210 cells by 3-deazauridine. Biochem Pharmacol. 1984;33(23):3739–42. doi: 10.1016/0006-2952(84)90034-0 6095859

[23] Moriconi WJ, Slavik M, Taylor S. 3-Deazauridine (NSC 126849): An interesting modulator of biochemical response. Invest New Drugs. 1986;4(1):67–84. doi: 10.1007/BF00172020 2422137

[24] Kang GJ, Cooney DA, Moyer JD, Kelley JA, Kim HY, Marquez VE, et al. Cyclopentenylcytosine triphosphate. Formation and inhibition of CTP synthetase. J Biol Chem. 1989;264(2):713–8. 2910861

[25] Toots M, Yoon J-J, Hart M, Natchus MG, Painter GR, Plemper RK. Quantitative efficacy paradigms of the influenza clinical drug candidate EIDD-2801 in the ferret model. Transl Res. 2020;218:16–28. doi: 10.1016/j.trsl.2019.12.002 31945316 PMC7568909

[26] Okesli-Armlovich A, Gupta A, Jimenez M, Auld D, Liu Q, Bassik MC, et al. Discovery of small molecule inhibitors of human uridine-cytidine kinase 2 by high-throughput screening. Bioorg Med Chem Lett. 2019;29(18):2559–64. doi: 10.1016/j.bmcl.2019.08.010 31420268 PMC6719797

[27] Suzuki NN, Koizumi K, Fukushima M, Matsuda A, Inagaki F. Structural basis for the specificity, catalysis, and regulation of human uridine-cytidine kinase. Structure. 2004;12(5):751–64. doi: 10.1016/j.str.2004.02.038 15130468

[28] Krissinel E, Henrick K. Inference of macromolecular assemblies from crystalline state. J Mol Biol. 2007;372(3):774–97. doi: 10.1016/j.jmb.2007.05.022 17681537

[29] Zhang SM, Rehling D, Jemth A-S, Throup A, Landázuri N, Almlöf I, et al. NUDT15-mediated hydrolysis limits the efficacy of anti-HCMV drug ganciclovir. Cell Chem Biol. 2021;28(12):1693-1702.e6. doi: 10.1016/j.chembiol.2021.06.001 34192523

[30] Chou SW, Scott KM. Rapid quantitation of cytomegalovirus and assay of neutralizing antibody by using monoclonal antibody to the major immediate-early viral protein. J Clin Microbiol. 1988;26(3):504–7. doi: 10.1128/jcm.26.3.504-507.1988 2833529 PMC266321

[31] Akinci E, et al. Elucidation of remdesivir cytotoxicity pathways through genome-wide CRISPR-Cas9 screening and transcriptomics. bioRxiv. 2020.

[32] Lim S-Y, Guo Z, Liu P, McKay LGA, Storm N, Griffiths A, et al. Anti-SARS-CoV-2 activity of adamantanes in vitro and in animal models of infection. COVID. 2022;2(11):1551–63. doi: 10.3390/covid2110111 37274537 PMC10238102

[33] Cox RM, Wolf JD, Plemper RK. Therapeutically administered ribonucleoside analogue MK-4482/EIDD-2801 blocks SARS-CoV-2 transmission in ferrets. Nat Microbiol. 2021;6(1):11–8. doi: 10.1038/s41564-020-00835-2 33273742 PMC7755744

[34] Cox RM, Wolf JD, Lieber CM, Sourimant J, Lin MJ, Babusis D, et al. Oral prodrug of remdesivir parent GS-441524 is efficacious against SARS-CoV-2 in ferrets. Nat Commun. 2021;12(1):6415. doi: 10.1038/s41467-021-26760-4 34741049 PMC8571282

[35] Grant S, Bhalla K, Gleyzer M. Effect of uridine on response of 5-azacytidine-resistant human leukemic cells to inhibitors of de novo pyrimidine synthesis. Cancer Res. 1984;44(12 Pt 1):5505–10. 6208998

[36] Liu J, Li Y, Liu Q, Yao Q, Wang X, Zhang H, et al. SARS-CoV-2 cell tropism and multiorgan infection. Cell Discov. 2021;7(1):17. doi: 10.1038/s41421-021-00249-2 33758165 PMC7987126

[37] Maas BM, Strizki J, Miller RR, Kumar S, Brown M, Johnson MG, et al. Molnupiravir: Mechanism of action, clinical, and translational science. Clin Transl Sci. 2024;17(2):e13732. doi: 10.1111/cts.13732 38593352 PMC10851176

[38] Iwamoto M, Duncan KE, Wickremasingha PK, Zhao T, Liberti MV, Lemoine L, et al. Assessment of pharmacokinetics, safety, and tolerability following twice-daily administration of molnupiravir for 10 days in healthy participants. Clin Transl Sci. 2023;16(10):1947–56. doi: 10.1111/cts.13602 37526305 PMC10582664

[39] Girish V, Lakhani AA, Thompson SL, Scaduto CM, Brown LM, Hagenson RA, et al. Oncogene-like addiction to aneuploidy in human cancers. Science. 2023;381(6660):eadg4521. doi: 10.1126/science.adg4521 37410869 PMC10753973

[40] Bonagas N, Gustafsson NMS, Henriksson M, Marttila P, Gustafsson R, Wiita E, et al. Pharmacological targeting of MTHFD2 suppresses acute myeloid leukemia by inducing thymidine depletion and replication stress. Nat Cancer. 2022;3(2):156–72. doi: 10.1038/s43018-022-00331-y 35228749 PMC8885417

[41] Zhang SM, Desroses M, Hagenkort A, Valerie NCK, Rehling D, Carter M, et al. Development of a chemical probe against NUDT15. Nat Chem Biol. 2020;16(10):1120–8. doi: 10.1038/s41589-020-0592-z 32690945 PMC7610571

[42] Zhang SM, et al. Identification and evaluation of small-molecule inhibitors against the dNTPase SAMHD1 via a comprehensive screening funnel. iScience. 2024;:108907. 38318365 10.1016/j.isci.2024.108907 PMC10839966

[43] Yadav B, Wennerberg K, Aittokallio T, Tang J. Searching for drug synergy in complex dose-response landscapes using an interaction potency model. Comput Struct Biotechnol J. 2015;13:504–13. doi: 10.1016/j.csbj.2015.09.001 26949479 PMC4759128

[44] Vonrhein C, Flensburg C, Keller P, Sharff A, Smart O, Paciorek W, et al. Data processing and analysis with the autoPROC toolbox. Acta Crystallogr D Biol Crystallogr. 2011;67(Pt 4):293–302. doi: 10.1107/S0907444911007773 21460447 PMC3069744

[45] McCoy AJ, Grosse-Kunstleve RW, Adams PD, Winn MD, Storoni LC, Read RJ. Phaser crystallographic software. J Appl Crystallogr. 2007;40(Pt 4):658–74. doi: 10.1107/S0021889807021206 19461840 PMC2483472

[46] Emsley P, Lohkamp B, Scott WG, Cowtan K. Features and development of Coot. Acta Crystallogr D Biol Crystallogr. 2010;66(Pt 4):486–501. doi: 10.1107/S0907444910007493 20383002 PMC2852313

[47] Kovalevskiy O, Nicholls RA, Long F, Carlon A, Murshudov GN. Overview of refinement procedures within REFMAC5: Utilizing data from different sources. Acta Crystallogr D Struct Biol. 2018;74(Pt 3):215–27. doi: 10.1107/S2059798318000979 29533229 PMC5947762

[48] Rentoft M, Lindell K, Tran P, Chabes AL, Buckland RJ, Watt DL, et al. Heterozygous colon cancer-associated mutations of SAMHD1 have functional significance. Proc Natl Acad Sci U S A. 2016;113(17):4723–8. doi: 10.1073/pnas.1519128113 27071091 PMC4855590

[49] Ranjbarian F, et al. Isocratic HPLC analysis for the simultaneous determination of dNTPs, rNTPs and ADP in biological samples. Nucleic Acids Res, 2022;50(3):e18. 10.1093/nar/gkab1117 PMC8860589 34850106

