# Supplementary figures and images for: Uridine cytidine kinases govern molnupiravir bioactivation and anti-SARS-CoV-2 activity
Source: PLoS Pathog. 2026 May 29;22(5):e1014225. doi: 10.1371/journal.ppat.1014225 (PMC13245873; doi:10.1371/journal.ppat.1014225)

S1 Figure

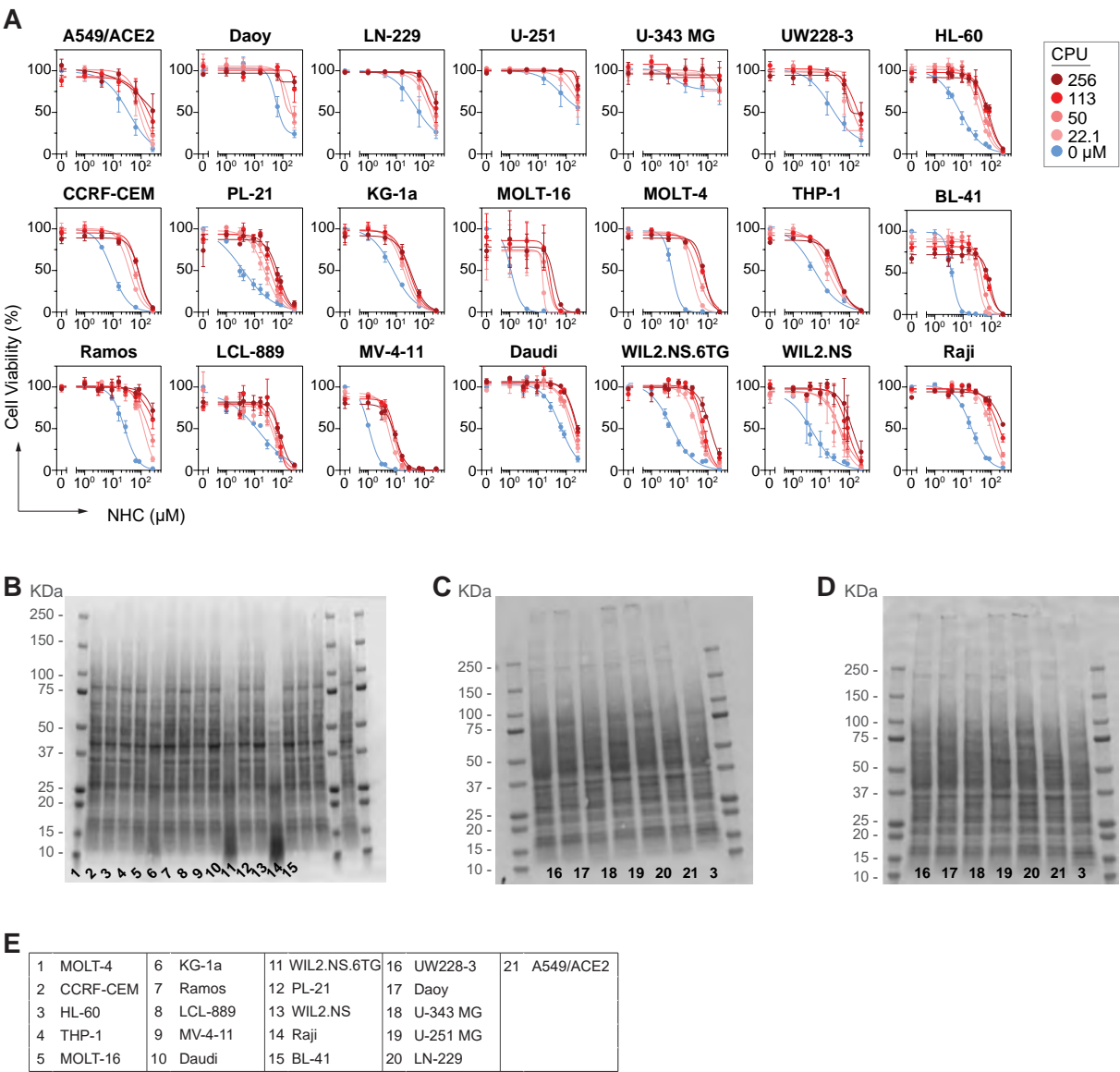

Supplement: S1 Fig — UCK inhibitor CPU dose-dependently antagonized the cytotoxic efficacy of NHC in multiple cell lines. Cells were treated with a dose-response matrix of NHC and CPU for 4 days before cell viability was determined using the resazurin reduction assay. Mean cell viability relative to DMSO control group ± SEM of n = 2–3 independent experiments are shown, which were further curve fitted using a nonlinear curve fitting model with variable slope (GraphPad Prism) to generate the cell viability curves. B-E. Ponceau red staining images of Western blot membranes shown in Fig 1E, to demonstrate cell lysates containing equal amounts of total proteins were analysed. In B, same membrane later probed for UCK1 and UCK2, generating Western blot image shown in Fig 1E left panel; in C and D, membranes later probed for UCK1 (C) and UCK2 (D), generating Western blot image shown in Fig 1E right panel. Sample identities are shown in E. (PDF) [file ppat.1014225.s001.pdf]

S2 Figure

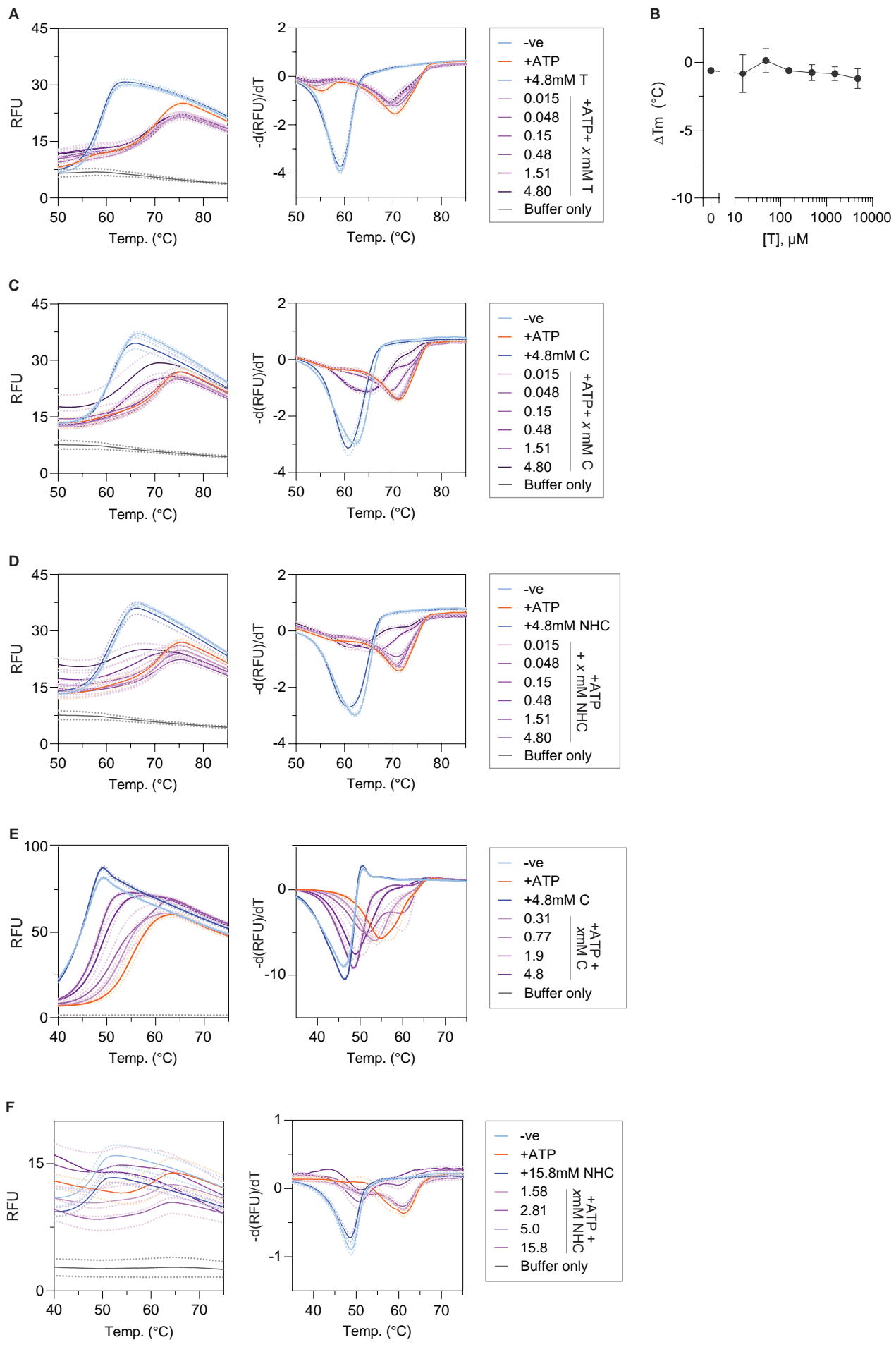

Supplement: S2 Fig — The non-substrate thymidine did not effectively engage recombinant UCK1, when applied up to 4.8 mM in the DSF assay. Recombinant UCK1 (5 µM) was incubated with indicated concentrations of thymidine or DMSO, in the presence or absence of ATP, before protein thermal stabilities were determined using the DSF assay. In A, left panel displays melting curves of a representative experiment performed in quadruplicate, where mean fluorescence signals (solid line) ± SEM (dashed line) are shown; right panel displays negative derivative (-dRFU/dT) of the melting curves in the left panel, where mean negative derivative values (solid lines) ± SEM (dashed lines) are shown. Protein melting temperatures (Tm) were determined as the local minima of the negative derivative curves, and changes of Tm compared to ATP-only group are shown as ΔTm in B, where mean ΔTm ± SEM of n = 4 independent experiments performed in triplicate to quadruplicate are shown. C-F. Melting profiles of recombinant UCK1 (C-D) and UCK2 (E-F) when incubated with NHC or C. Recombinant UCK proteins were incubated with increasing concentrations of NHC, C or DMSO, in the presence or absence of ATP, before protein thermal stabilities were determined using the DSF assay. Left panels, melting curves of a representative experiment performed in quadruplicate, where mean fluorescence signals (solid line) ± SEM (dashed line) are shown; right panel, negative derivative (-dRFU/dT) of the melting curves in the left panel, where mean negative derivative values (solid lines) ± SEM (dashed lines) are shown. The curves are supplemental to Fig 2E-F. (PDF) [file ppat.1014225.s002.pdf]

S3 Figure

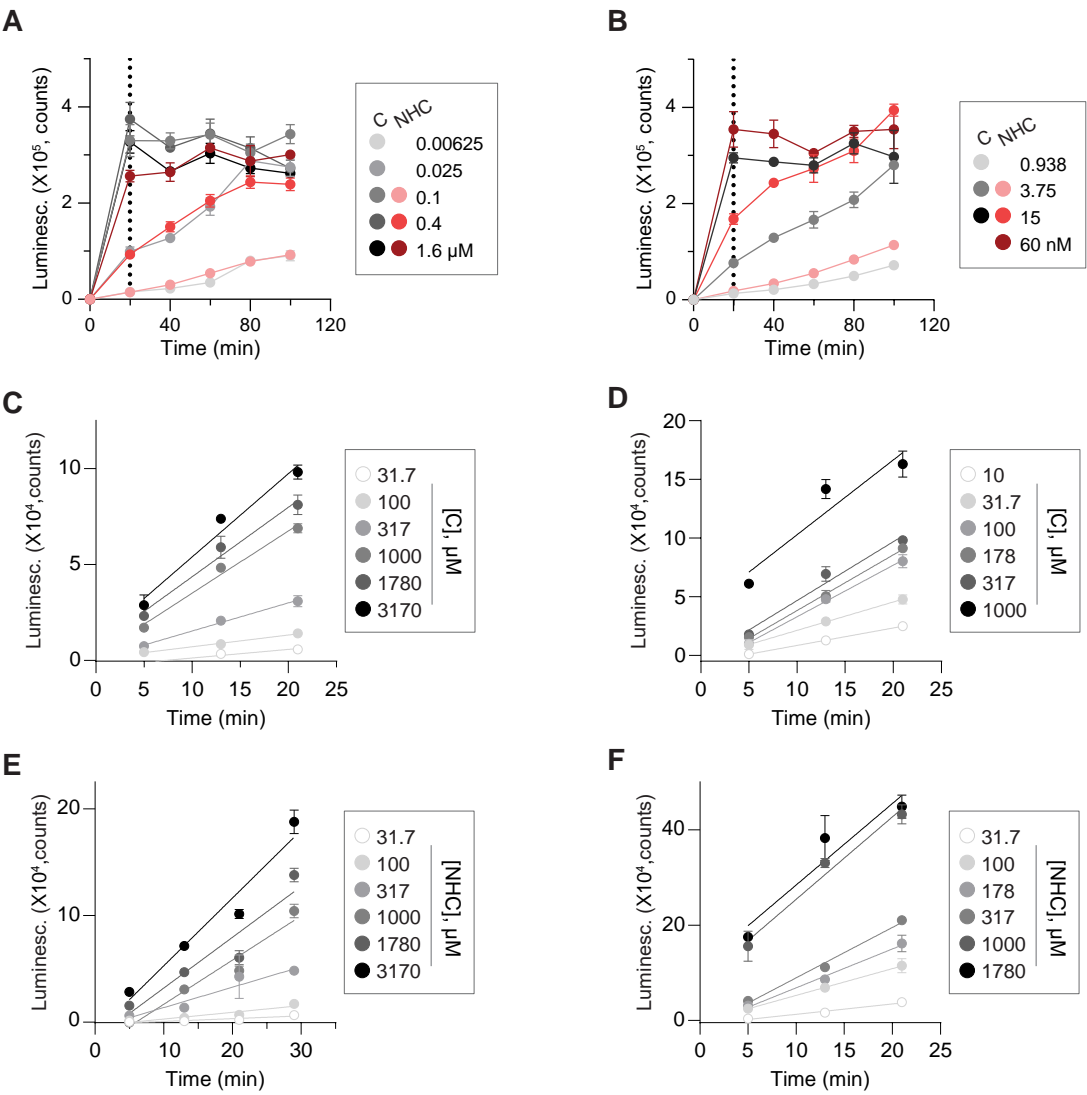

Supplement: S3 Fig — Titration of UCK1 (A) and UCK2 (B) when using cytidine or NHC as the substrate. Recombinant UCK1 and UCK2 of varying concentrations were incubated with 200 µM or 100 µM substrates, respectively. Reactions were allowed to proceed for indicated periods before the addition of ADP-Glo reagent and kinase detection reagent, with incubation of 1 hour between each addition. Luminescence measurement was then done on a Hidex plate reader. Mean luminescence signals ± SEM of independent experiments performed in triplicate are shown. C-D. Kinetic studies of phosphorylation of cytidine by recombinant UCK1 (C) and UCK2 (D), supplementary to Fig 3A and 3B, respectively. The reaction was linear under the specified conditions for the duration of the experiment. Mean luminescence ± SEM of a representative experiment performed in triplicate are shown, which were subsequently used to determine V0 and reaction kinetic parameters. E-F. Kinetic studies of phosphorylation of NHC by recombinant UCK1 (E) and UCK2 (F), supplementary to Fig 3C and 3D, respectively. The reaction was linear under the specified conditions for the duration of the experiment. Mean luminescence ± SEM of a representative experiment performed in triplicate are shown, which were subsequently used to determine V0 and reaction kinetic parameters. (PDF) [file ppat.1014225.s003.pdf]

S4 Figure

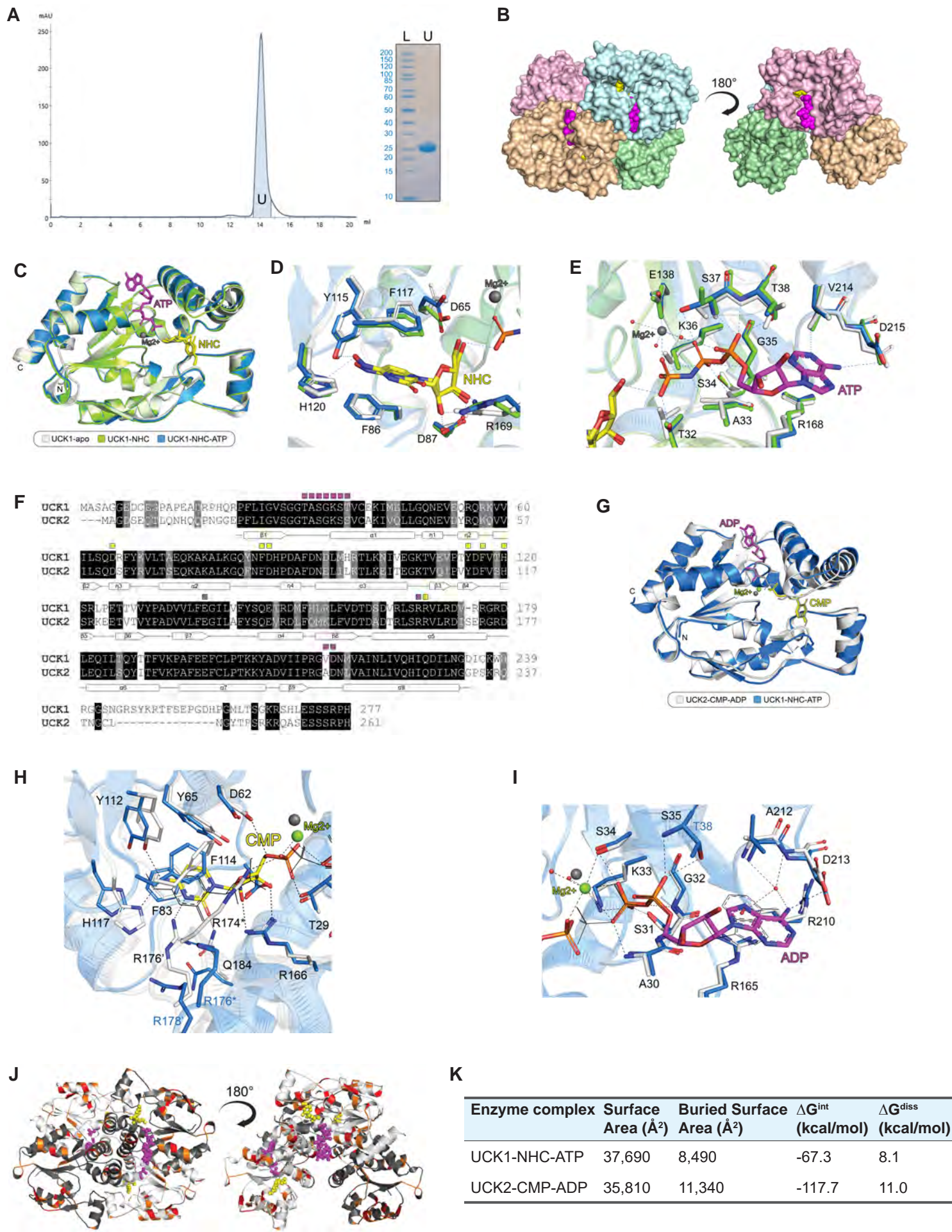

Supplement: S4 Fig — Tetramerization of the purified recombinant UCK1 (amino acids 21–235). Left: Chromatogram from Gel filtration chromatography run performed using a Superdex200 increase 10/300 GL column (Cytiva). The single peak (labelled U) is consistent with the expected size of the hUCK1 tetramer. Right: Corresponding SDS-PAGE gel analysis. 20 micrograms of protein was loaded onto the gel. L: PageRuler unstained protein ladder (Thermo Fisher Scientific). U: UCK1 (amino acids 21–235) protein sample. B. Multiple views of human UCK1-NHC-AMPPNP tetramer. Surface representation of the UCK1-NHC-AMPPNP tetramer where individual monomers are colored light pink, light cyan, light green and wheat. NHC and ATP are depicted as spheres colored yellow and magenta, respectively. C. Cα-atom superpositions of UCK1-NHC (green) with UCK1-NHC-AMPPNP (blue) and apo-UCK1 (white, PDB ID: 2jeo). The magnesium ion from UCK1-NHC-AMPPNP is shown as a gray sphere. NHC and ATP are depicted as sticks colored yellow and magenta, respectively. D-E. Comparison of NHC (D) and ATP (E) binding sites in UCK1-NHC and UCK1-NHC-AMPPNP with apo-UCK1. Amino acid side chains are shown as sticks; C atoms are coloured according to the color scheme shown in panel C, O atoms colored red, N atoms colored blue, and P atoms colored orange. The magnesium ion from UCK1-NHC-AMPPNP is shown as a gray sphere. NHC and AMPPNP are depicted as sticks colored yellow and magenta, respectively. Hydrogen bonds from the UCK1-NHC-AMPPNP structure are depicted as dashed lines. F. Amino acid sequence alignment of human UCK1 (UniProt ID: Q9HA47) and human UCK2 (UniProt ID: Q9BZX2). Identical residues are shaded black, while grey shading indicates amino acids with conserved physicochemical properties. The secondary structure annotation of UCK1-NHC-AMPPNP is shown below the alignment. Amino acids from UCK1-NHC-AMPPNP required for NHC, ATP or magnesium binding are indicated by boxes above the alignment coloured yellow, magenta or dark grey, respectiv [file ppat.1014225.s004.pdf]

S5 Figure

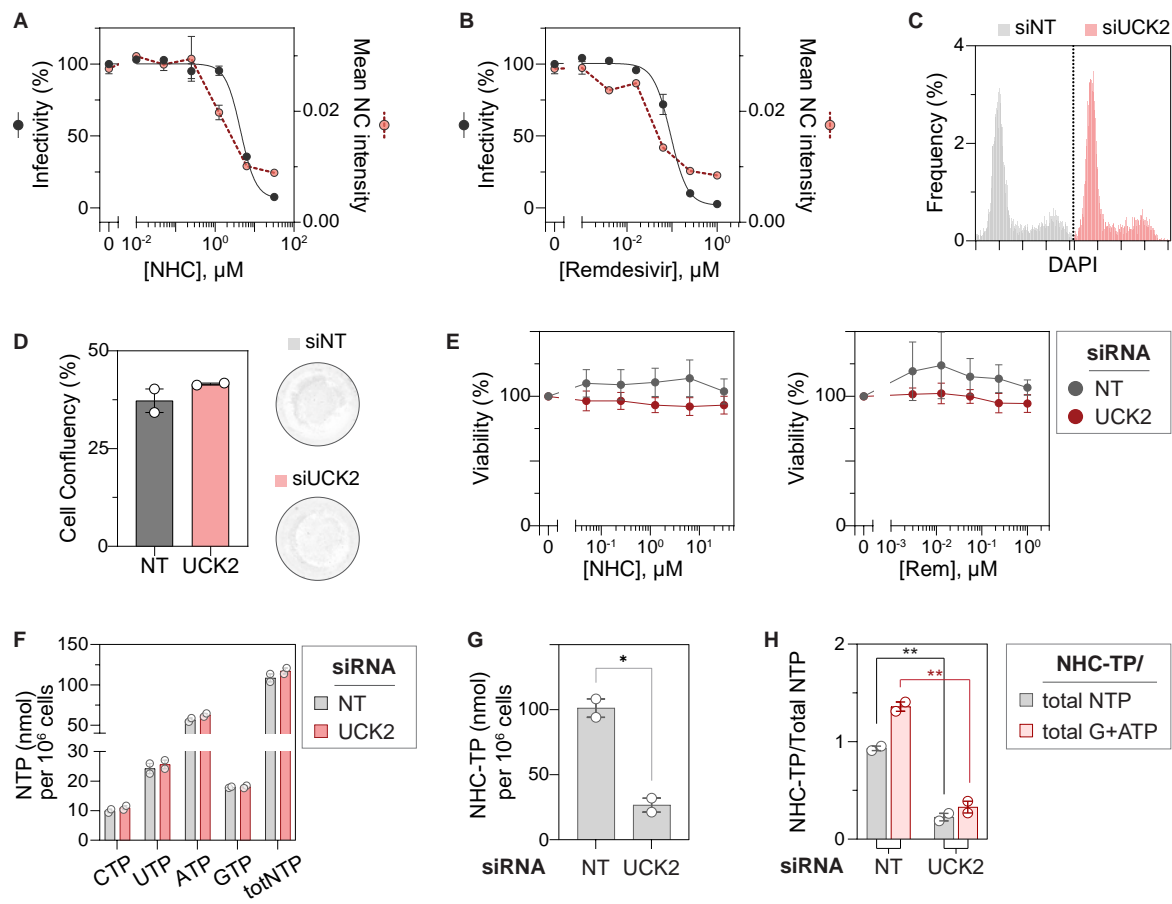

Supplement: S5 Fig — The high-content imaging-based SARS-CoV-2 infectivity assay produced antiviral efficacies of NHC (A) and Remdesivir (B) that are comparable to past studies. A549/ACE2 cells were infected overnight with SARS-CoV-2 at an MOI of 0.07 in the presence of antivirals or the diluent control DMSO, before cells were fixed and stained for viral nucleocapsid protein (NC) and nuclei using NC-specific antibody and DAPI, respectively. Infectivity was estimated as the ratio of NC/DAPI double-positive cells over DAPI single-positive cells, and further normalized to DMSO-only control group. The resulting relative infectivity (%) data were then curve fitted using a nonlinear curve fitting model with variable slope (GraphPad Prism) to generate the antiviral EC50. The estimated infectivity agreed with mean NC intensity in cells stained positive for DAPI, showcasing the robustness of the automated cell profiler pipeline for identifying NC/DAPI double-positive cells. Mean infectivity relative to DMSO-only control group ± SEM, as well as mean NC intensity/cells/well, of a representative experiment performed in duplicate are shown. C. UCK2 knockdown did not affect A549/ACE2 cell cycle progression. A549/ACE2 cells were transfected with UCK2-specific (siUCK2) or non-targeting control (siNT) siRNA. Four days post-transfection, cells were fixed and stained for DAPI, followed by high-content imaging acquisition and DAPI signal analysis using CellProfiler. Frequency distribution analysis of DAPI signals were then done using GraphpadPrism. D. UCK2 knockdown did not affect A549/ACE2 cell proliferation. A549/ACE2 cells were transfected with UCK2-specific (siUCK2) or non-targeting control (siNT) siRNA. One day post-transfection, cells were re-seeded and allowed to proliferate for four days, before cell growth was determined via the confluence analysis function on a Tecan Spark Cyto imaging cytometer. Left panel, mean cell confluence ± SEM of n = 2 independent experiments; right panel, representative [file ppat.1014225.s005.pdf]

S6 Figure

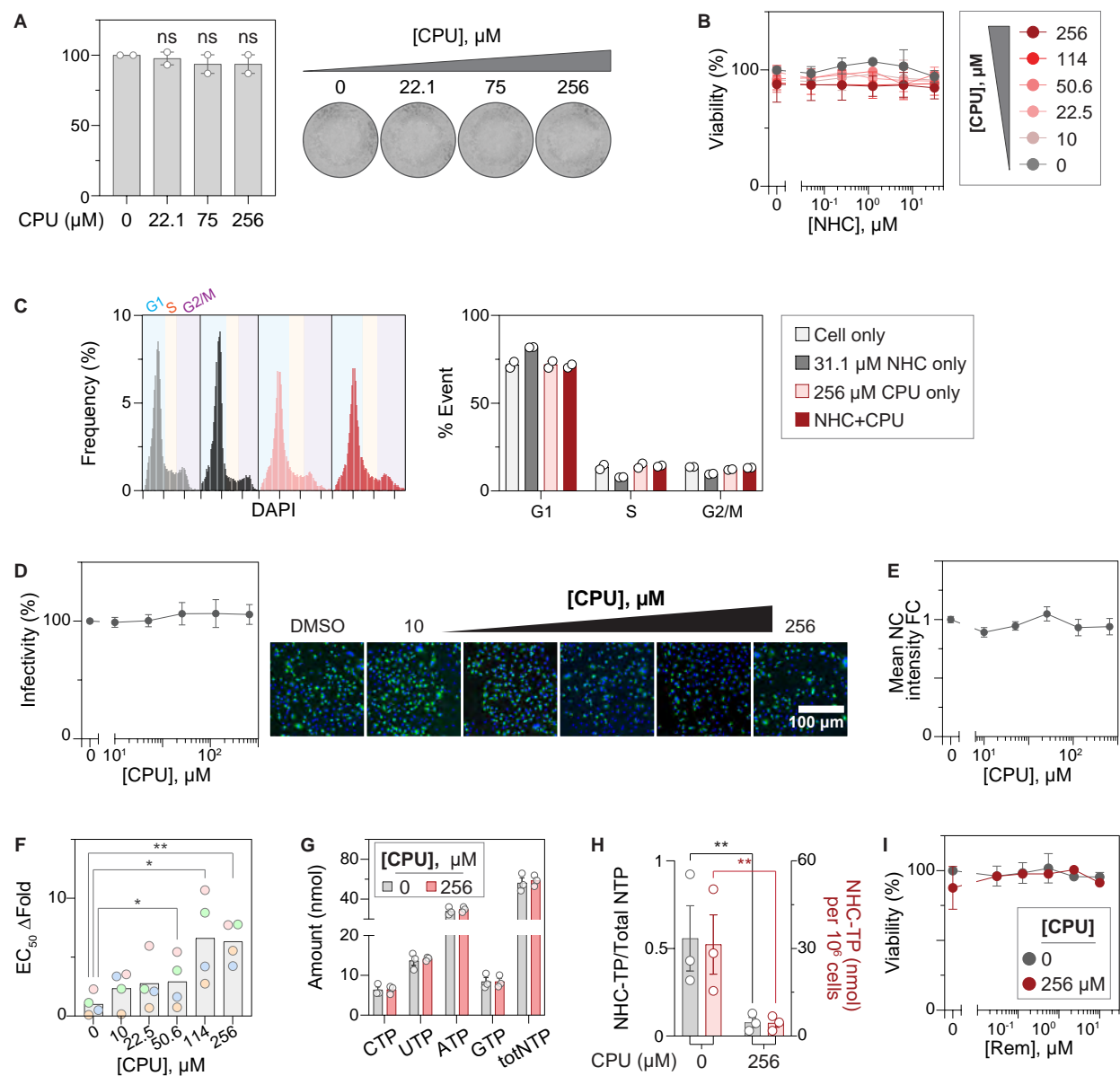

Supplement: S6 Fig — UCK inhibition via CPU did not affect A549/ACE2 cell proliferation, shown via confluence analysis. A549/ACE2 cells were seeded overnight and then treated with indicated concentrations of CPU or the diluent control DMSO for four days before cell growth was assessed using the confluence analysis function on a Tecan Spark Cyto imaging cytometer. Left panel, mean cell confluence relative to DMSO-only group ± SEM of n = 2 independent experiments; right panel, representative cell growth pictures used for confluence analysis. Dunnett’s multiple comparisons tests were performed across treatment groups [0 µM CPU vs. 22.1 µM CPU, p = 0.9762, q = 0.3173, DF = 4; 0 µM CPU vs. 75 µM CPU, p = 0.7345, q = 0.8690, DF = 4; 0 µM CPU vs. 256 µM CPU, p = 0.7345, q = 0.8690, DF = 4], where ns signifies statistical insignificance (p > 0.05). B-C. During the course of the 1-day viral infectivity assay, NHC and/or CPU did not affect cell viability (B) or cell cycle progression (C). A549/ACE2 cells were treated overnight with a concentration matrix of NHC and CPU, at the same concentrations as in viral infectivity assay shown in Fig 6. Following the treatment, in B, cell viabilities were determined using the resazurin reduction viability assay. Relative viability normalized to cells treated with DMSO only ± SEM of n = 2 independent experiments are shown. In C, cell cycle was determined following the treatment with highest concentrations of NHC and/or CPU, where DAPI-stained cells were imaged by high-content imaging followed by DAPI intensity analysis via CellProfiler. Left panel, DAPI-intensity histograms with gating strategy of a representative experiment; right panel, mean percentages of cells in different cell cycle phases ± SEM of n = 2 independent experiments, together with individual experiment values, are shown. D-E. CPU alone did not inhibit SARS-CoV-2 infectivity. A549/ACE2 cells were infected with SARS-CoV-2 overnight with or without CPU, before viral infectivity and nucleocapsid [file ppat.1014225.s006.pdf]

S7 Figure

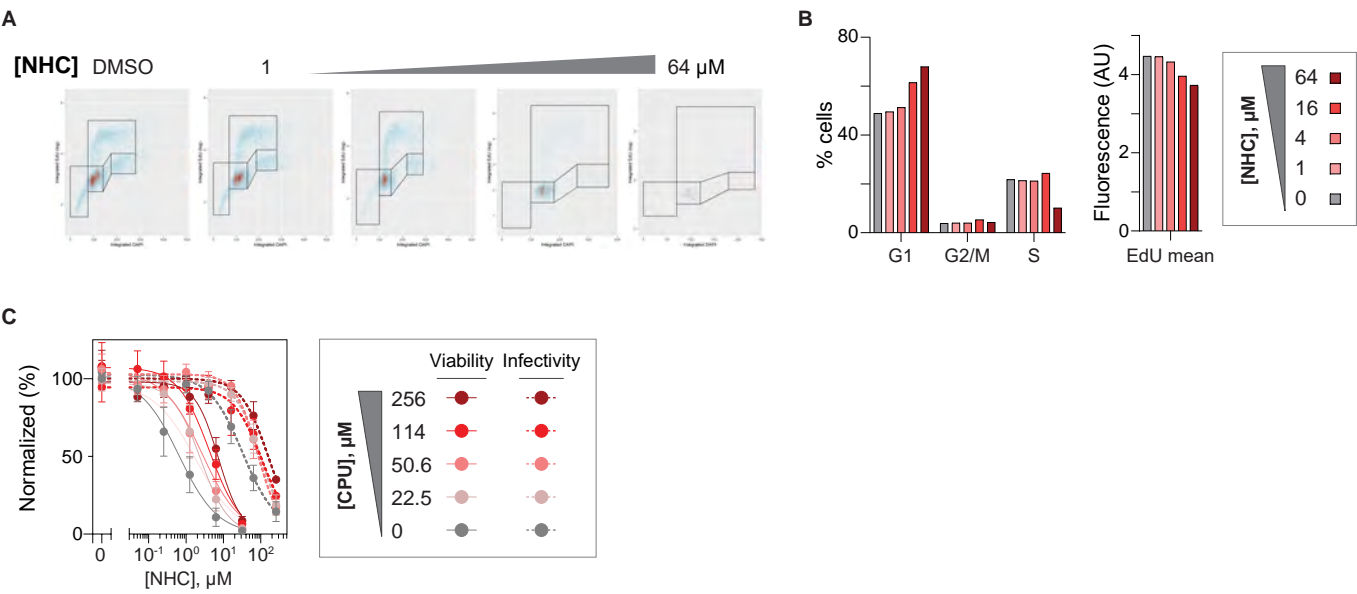

Supplement: S7 Fig — Four day-treatment with NHC induced S phase collapse and G1 arrest in A549/ACE2 cells. A549/ACE2 cells were treated with increasing concentrations of NHC for four days before cell cycles were analysed via immunofluorescence staining for EdU incorporation and DAPI followed by high-content imaging. In A, representative plots with gating strategy illustrated. In B, percentage of cells in each cell cycle (left panel) and mean integrated EdU signal of cells in S phase (right panel) of a representative experiment, illustrating that NHC dose-dependently induced collapse of EdU-incorporating S phase cells concurrent to G1 arrest. C. Viral infectivity inhibition curves of SARS-CoV-2 infection in A549/ACE2 cells, and the viability curves of A549/ACE2 cells generated using the same dose-response matrix of CPU and NHC. In the viral infectivity assay, A549/ACE2 cells were infected with SARS-CoV-2 in the absence or presence of CPU and/or NHC. One day post-infection, viral infectivity was determined via staining the cells with viral nucleocapsid protein followed by high-content imaging analysis. Mean relative infectivity normalized to the DMSO-only group ± SEM of n = 4 independent experiments performed in duplicate are shown. Meanwhile the effect of NHC on cell viability was determined with the same NHC/CPU matrix but a prolonged four-day drug treatment, followed by resazurin viability reduction assay. Mean viability relative to DMSO group ± SEM of n = 2–4 independent experiments are shown. Both viability and infectivity data were further curve fitted using a nonlinear curve fitting model with variable slope (GraphPad Prism) to generate the antiviral EC50 and growth inhibitory CC50 values of NHC, which were further used to calculate EC50 (24h)/CC50 (96h) as shown in Fig 7D. (PDF) [file ppat.1014225.s007.pdf]

S8 Figure

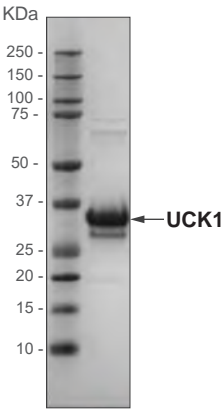

Supplement: S8 Fig — Purified recombinant UCK1 protein (4 µg) was subject to SDS-PAGE followed by Coomassie brilliant blue staining, demonstrating protein purity. (PDF) [file ppat.1014225.s008.pdf]

S9 Figure

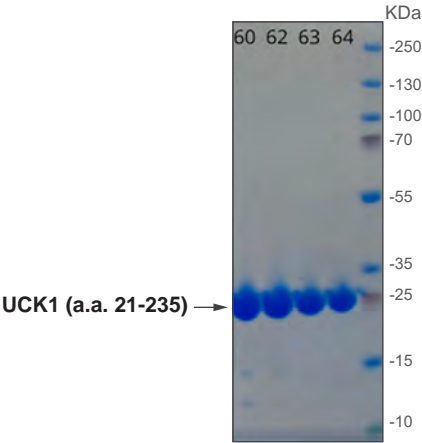

Supplement: S9 Fig — Recombinant UCK1 (amino acids 21–235) fractions post-gel filtration(fraction number indicated in the picture) was analysed by SDS-PAGE followed by Coomassie brilliant blue staining, demonstrating high protein purity. The fractions shown were then pooled for protein X-ray crystallography studies. (PDF) [file ppat.1014225.s009.pdf]

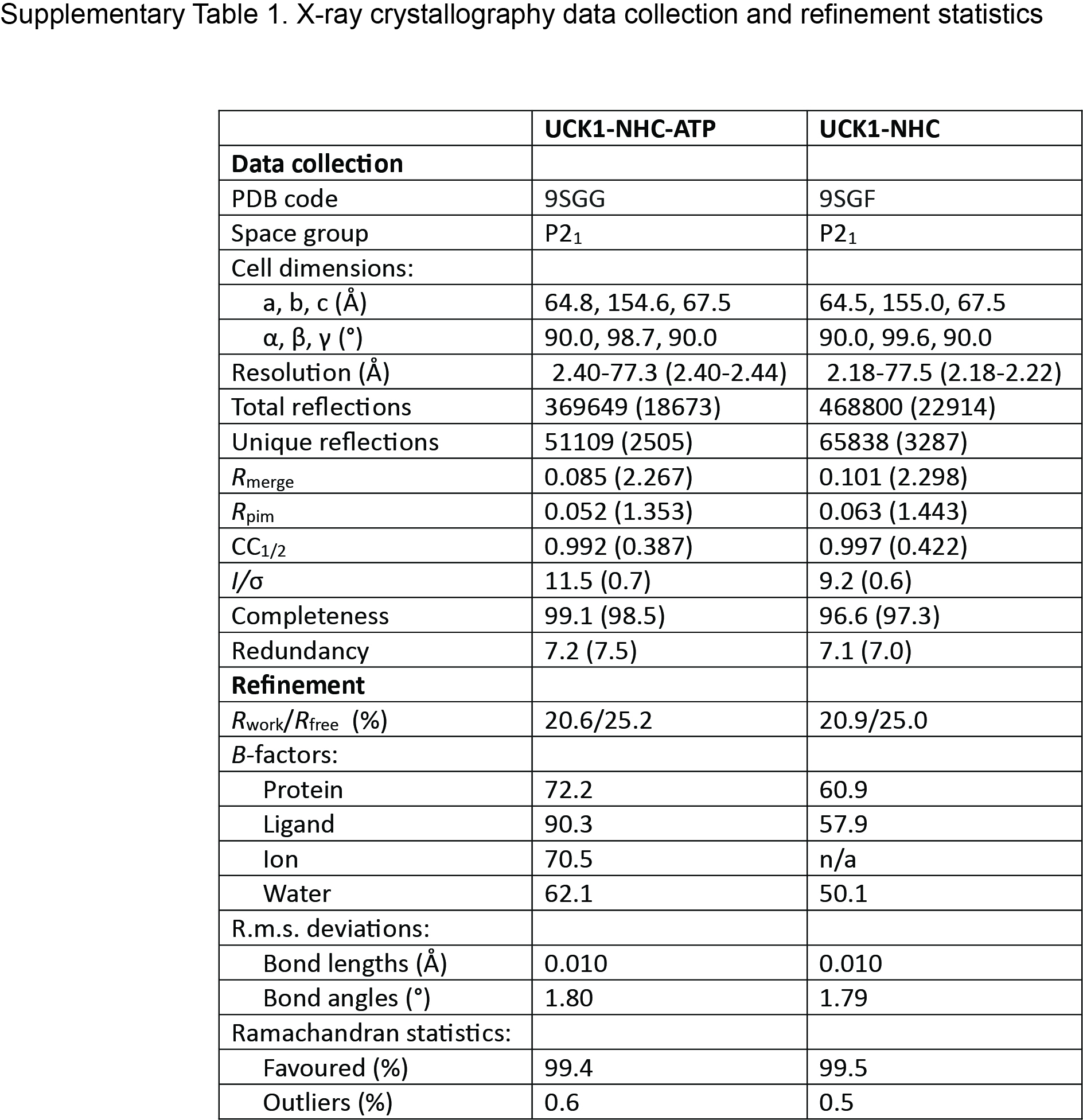

Supplement: S1 Table — (JPG) [file ppat.1014225.s010.jpg]
