# Supplementary material for: Uridine cytidine kinases govern molnupiravir bioactivation and anti-SARS-CoV-2 activity
Source: PLoS Pathog. 2026 May 29;22(5):e1014225. doi: 10.1371/journal.ppat.1014225 (PMC13245873; doi:10.1371/journal.ppat.1014225)
Supplement: S1 Raw Image — (PDF) [file ppat.1014225.s011.pdf]

Uncropped WB blots

Fig1E

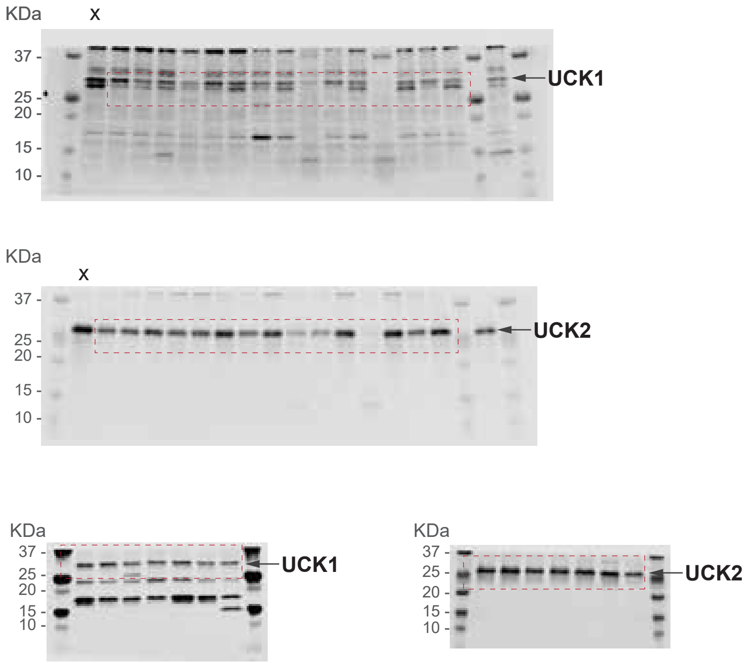

Fig5B

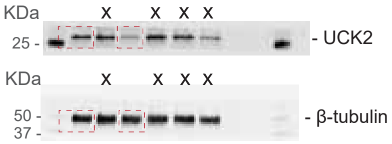

Un-cropped Western Blot images for experiments presented in the specified figures. Images were captured on an Odyssey Fc Imaging System (Li-Cor Biosciences). Lanes included in the specified figures are indicated by the red dashed rectangles and lanes not included are labeled with 'X' marks.
